# Movement Patterns of Laying Hens Across Production Stages in a Commercial Aviary System

**DOI:** 10.3390/ani16213533

**Published:** 2026-11-09

**Authors:** Mina Omrani, Timothy Schaerf, Terence Zimazile Sibanda, Isabelle Ruhnke, Mitchell Welch

**Affiliations:** 1 School of Science & Technology, University of New England, Armidale, NSW 2351, Australia; tschaerf@une.edu.au (T.S.); terence.sibanda@feedworks.com.au (T.Z.S.); mwelch8@une.edu.au (M.W.); 2 Feedworks Pty Ltd., Romsey, VIC 3434, Australia; 3 Faculty of Veterinary Medicine, Livestock Clinic, Division of Poultry, Freie Universität Berlin, 14163 Berlin, Germany; isabelle.ruhnke@fu-berlin.de; 4 Animal Science, School of Environmental and Rural Science, University of New England, Armidale, NSW 2351, Australia

**Keywords:** RFID, laying hens, aviary housing, movement sequences, transition rates, self-loops, precision livestock farming

## Abstract

We characterised radio-frequency identification (RFID) transitions among monitored areas of a commercial multi-tier aviary using a four-stage cohort of 2162 Lohmann Brown hens. The analysis used 204 retained dates from 21 November 2016 to 15 November 2017, approximately 18–70 weeks of age according to the supplied production records. Source bouts shorter than 10 s were excluded, and successive same-area bouts separated by at most 10 s were merged. Self-loops comprised 73.9% of 23,756,706 recorded transitions and were concentrated at the feeders. Pooled non-looping rates were 9.65, 13.54, 17.77 and 15.10 transitions per detected hen-day during pre-, peak-, late- and end-laying. Lower feeders formed the strongest cross-area connections, with pop-hole links more prominent later. Restricting existing links to the six indoor feeder/nest-box locations reduced the peak-to-late increase from 31.2% to 2.7%. A higher-coverage cohort and timing sensitivities retained the same ordering of stage rates. Adding eligible cross-midnight non-looping links increased counts by less than 0.9% per stage. These descriptive results separate repeated detection from cross-area adjacency, while showing that contrast magnitudes depend on the locations represented. Age, season, management and monitoring coverage changed together; the records do not identify their separate effects or verify continuous occupancy or outdoor journeys.

## Simple Summary

We examined automated location records from 2162 hens in five sections of one commercial aviary during four production stages. We separated repeated detections in one area from transitions between different monitored areas. Nearly three quarters of recorded transitions were repeated same-area detections, mainly at the feeders. Lower feeders commonly connected different monitored locations, and links between lower feeders and the pop-hole were more prominent later in the recording period. The size of the stage contrasts depended on which locations were included, and monitoring coverage also changed over time. These patterns describe the observed records and help identify where more complete monitoring would be useful. They do not establish that age, diet or an outdoor feeder caused a change in movement.

## 1. Introduction

Cage-free systems, including multi-tier aviaries, are increasingly used as alternatives to conventional cages, with the aim of improving laying-hen welfare by providing greater space, access to key resources, and opportunities to perform motivated behaviours. However, these systems also introduce management, health and welfare challenges [1,2]. Commercial multi-tier aviaries are structurally complex: hens can move vertically among tiers containing feeders, nest boxes, and perches, and horizontally among functional areas, including pop-holes and outdoor ranges. Their movement and space use may also vary across the day and production cycle [3,4]. Patterns of resource access are relevant to suitable nest-site use, competition for nest boxes, crowding and smothering, collision-related injuries, feeding routines and daily activity budgets [5,6,7,8,9]. Quantifying how hens use different components of commercial aviaries throughout the production cycle is therefore important for understanding movement structure and informing flock and housing management.

Radio-frequency identification (RFID) systems are widely used to collect automated animal-location data. A typical system consists of a tag attached to the animal, antenna receivers that register the tag within a detection field and a central unit that records the resulting spatial and temporal information [10,11]. RFID enables long-term tracking of many individuals without requiring line of sight, making it well suited to structurally complex environments such as commercial aviaries. In poultry research, RFID has been used to investigate space-use patterns, resource visits, and ranging behaviour in laying hens [12,13,14,15]. To date, RFID tracking is primarily a research tool in farming systems; it is not routinely used in day-to-day commercial egg or meat production, but it enables fine-scale behavioural measurement at the flock scale.

A growing body of research has examined how hens distribute themselves within complex housing systems and how these patterns relate to welfare and health. Rufener et al. [16,17] reported consistent individual differences in mobility and location use and identified associations between mobility and keel-bone outcomes. Validation research has demonstrated the potential of RFID for monitoring movement among aviary levels, while also showing that tracking performance depends on the spatial resolution examined and may be lower for specific antennas than for broader tier-level locations [18]. Welch et al. [19] subsequently used RFID-derived location-duration features from a broader dataset that included the flock examined here in machine-learning models of health outcomes. Together, these studies demonstrate the potential value of individual location records while also emphasising the importance of clearly defining what the RFID system measures.

RFID has also been used to monitor activity, infer behavioural states and study spatial or social organisation in other animals [20,21,22,23,24,25,26]. These applications illustrate the value of time-ordered records, but their interpretation depends on the detection system and the spatial resolution represented.

RFID detections indicate that a tag was registered within an antenna field; they do not directly identify the behaviour performed or provide a continuous trajectory between monitored locations [27]. Source RFID records can also include very short bouts and fragmentation of same-area detection across brief interruptions. Temporal criteria are therefore commonly used to determine which records are retained and when successive records should be treated as part of the same area-level bout [28]. These analytical choices can affect the resulting sequence and, consequently, the number and type of derived transitions. Transparent reporting and evaluation of the temporal criteria are particularly important when consecutive bouts assigned to the same monitored area are retained as self-loops. Once constructed, time-ordered location sequences can be represented as directed transitions or movement networks to examine routing among monitored areas [29]. However, relatively few studies in commercial aviaries have examined the information retained by same-area self-loops alongside transitions between different functional areas.

The present analysis extends two strands of work from the same research programme. Welch et al. [19] summarised daily durations at feeders, nest boxes and the range across production stages for health prediction. Those features describe how much recorded time was associated with each resource, but do not retain which area immediately preceded or followed another. Ruhnke et al. [30] reviewed movement monitoring and illustrated pathways among resources, while Sibanda et al. [31] examined associations between range use and aviary use during early lay. Directed representations retain the order of recorded locations [29]. Here, we quantify directed adjacency across four retained-date stages and separately report repeated same-area detection. This distinguishes, for example, an increase in feeder–pop-hole links from an increase in repeated feeder detections, even when the total recorded activity rises. The contribution is a descriptive analysis of sequence structure and its sensitivity to observation choices, rather than a new tracking technology or a validated behavioural classifier.

We asked how recorded transition rates and the relative prominence of cross-area routes varied among production stages, and what was gained by separating same-area self-loops from transitions between different areas. A self-loop (A→A) is a pair of successive cleaned bouts assigned to one monitored area; a non-looping transition (A→B) joins different monitored areas. Throughout the paper, these are properties of recorded sequences. They do not establish continuous occupancy, a physical departure and return, the path between detections or a completed outdoor visit. Hens were rearranged between pre- and peak-laying, and subsequent conditions were represented by individual housing sections. Stage and section comparisons are therefore descriptive associations within one flock.

## 2. Materials and Methods

### 2.1. Data Collection and Study System

This secondary study analysed RFID records from a commercial free-range flock of Lohmann Brown laying hens (*Gallus gallus domesticus*). A total of 3125 hens were fitted with RFID leg bands at approximately 16 weeks and initially allocated across five monitored housing sections (pens P1–P5), approximately 625 hens per section, within a commercial NATURA Step multi-tier aviary (Big Dutchman, Holland, MI, USA). The project calendar records placement on 7 November 2016. The retained observations span 21 November 2016–15 November 2017, with an estimated age range of approximately 18–70 weeks from the supplied production records; the age reference is described below. Indoor and outdoor stocking densities were 9 hens/m^2^ and 1500 hens/ha, respectively [31].

At placement, the 3125 tagged hens were selected from approximately 40,000 hens in the commercial flock. The original investigators described the initial selection and assignment to the monitored sections as random, although the randomisation sequence was not documented in the records available for this secondary analysis. The number of tagged hens was determined by pen and pop-hole capacity at the stated stocking density; no advance power calculation was performed. Farm managers and staff provided routine care under standard commercial procedures. No blinding was used; the hens’ subsequent range-use categories were unknown at initial placement.

**Figure 1:**
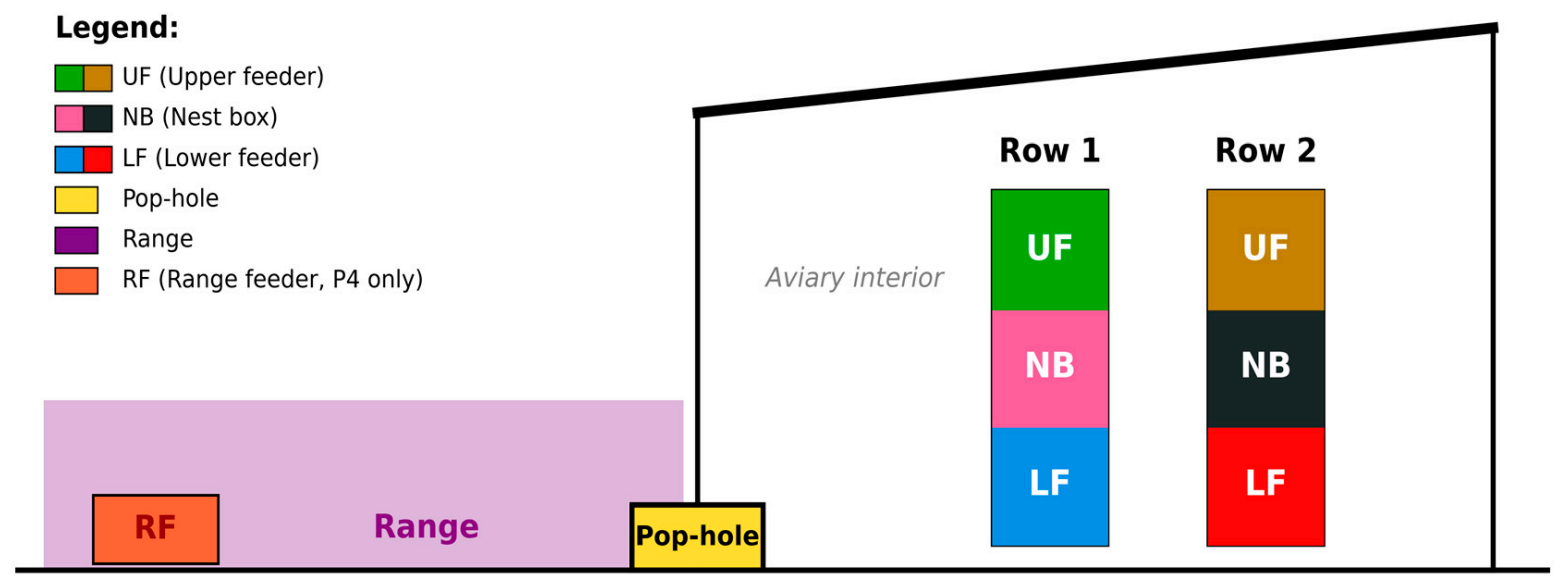
Schematic representation of the monitored functional areas in the commercial aviary system. Details of the aviary system and RFID installation were reported previously [31]. Each section comprised two aviary rows (Rows 1 and 2), with three functional areas in each row: lower feeder (LF), nest box (NB), and upper feeder (UF). Additional locations included the pop-hole, outdoor range, and an outdoor range feeder (RF) present only in P4. Paired antennas were installed on the inner and outer sides of each pop-hole; detections from both sides were mapped to a common Pop-hole node for the present analysis. Location labels used throughout the analysis follow the format Row:Area (e.g., R1:LF = Row 1, lower feeder; R2:NB = Row 2, nest box). Row designations were assigned for the present analysis using the antenna-management map: R1 represents the row nearer the pop-hole and R2 the row farther from the pop-hole.

As shown in Figure 1, each housing section contained two parallel aviary rows. The row nearer the pop-hole and range-facing side was designated Row 1 (R1), while the parallel row positioned farther from the pop-hole was designated Row 2 (R2). Each row contained three monitored functional areas: a lower feeder (LF), nest box (NB), and upper feeder (UF). Additional monitored locations included the pop-hole and outdoor range. The installation included Range antennas in the five sections, but their recorded coverage differed over time; P4 alone contained an outdoor range feeder (RF). Each pop-hole extended across the full 3.6-m width of its housing section.

The project calendar records progressive pop-hole opening from 14 November 2016 and full opening from 21 November 2016. Thereafter, usual range access was approximately 09:00–20:00 h, subject to weather and farm management. Production records list shorter light durations during pre-laying and a later 16-h schedule. The fixed 04:00–19:59 h window used in the recording audit was an operational coverage window; it should not be equated with the actual light period on every pre-laying date.

The RFID system comprised Speedway R420 readers, Monza R6 ultra-high-frequency RFID tags operating at 915 MHz (Impinj, Seattle, WA, USA), and ClearStream RFID software (Portable Technology Solutions, Calverton, NY, USA; https://www.clearstreamrfid.com/; acquisition software version not documented in the available records). Antennas were installed along feeder chains and nest-box entrances. Paired readers were positioned around the inner and outer sides of the pop-hole interface. Both were mapped to one Pop-hole node in this analysis, so the resulting transitions do not identify crossing direction. Each source RFID record contained the hen identifier, antenna identifier, and entry and exit timestamps. The entry time represented the first valid read within an antenna-level event, and the exit time represented the final valid read; intermediate reads had already been filtered by the RFID software. Further details of the RFID system and its validation have been reported previously [31]. Database timestamps were analysed as supplied, without applying a clock offset or daylight-saving adjustment.

The four stage names and nominal age bands followed Welch et al. [19]: pre-laying, 18–22 weeks; peak-laying, 23–33 weeks; late-laying, 34–54 weeks; and end-laying, 55–74 weeks. The predefined recording windows were 21 November–21 December 2016, 5 January–9 March 2017, 13 March–2 August 2017 and 4 August–15 November 2017, respectively. These nominal bands do not mean that every age in each band was represented. After allocation and coverage exclusions, the first and last retained dates were 21 November–15 December, 5 January–9 March, 16 March–2 August and 4 August–15 November, respectively (Table S7).

Age reporting was reconciled with the supplied daily production records. The first 284 available age-in-days values progressed consistently with calendar time, and the corresponding relation was consistent with all 379 recorded weekly-age labels. It gives approximate retained-age ranges of 18.4–21.9, 24.9–33.9, 34.9–54.7 and 55.0–69.7 weeks across the four stages. These are record-derived estimates, not ages established from a verified hatch date. The daily counter omitted 14 calendar days after an August–September recording gap; it was not used to redefine stages. The nominal week-74 endpoint therefore describes the broader stage definition, not the endpoint of the retained RFID observations (Supplementary Section S8.1).

During the initial pre-laying period (18–21 weeks of age), hens were classified as stayers, roamers or rangers according to their RFID-recorded range-use behaviour, following the procedure described by Sibanda et al. [32]. For the flock analysed here, the official flock-management records showed that hens were weighed and rearranged on the night of 19 December 2016, at 22 weeks of age. The post-rearrangement allocation was P1 = stayers, P2 = roamers, P3 = rangers maintained under standard conditions, P4 = rangers provided with an outdoor range feeder and P5 = rangers receiving the modified diet. RFID monitoring continued throughout the study, subject to the recording-date retention criteria described below. The same management records documented no subsequent transfers among housing sections or additions of tagged hens.

### 2.2. Recording Coverage and Retained-Date Selection

The RFID system was intended to operate continuously for 24 h, but some source dates were missing or had restricted recording spans. Coverage was evaluated separately by date and section before cleaning. The criteria used observed records as operational indicators and did not independently verify hardware uptime.

A date was retained only when it was compatible with the relevant allocation phase and all five housing sections simultaneously satisfied the following criteria: (i) records were present in every section; (ii) the interval between the first and last recorded event in each section was at least 20 h; (iii) every hourly interval in the fixed 04:00–19:59 h audit window contained recorded events; (iv) the number of records in each section was at least 75% of the median daily record count for that production-stage × section combination; and (v) the number of active antennas in each section was at least 80% of the corresponding stage × section median. These were section-wide recording-system criteria and did not require every individual hen to be detected for 20 h.

The rearrangement date, 19 December 2016, was excluded. To avoid mixing the pre- and post-rearrangement allocations within the pre-laying stage, dates after rearrangement were not included in the pre-laying analysis. Application of the recording-quality criteria retained 204 dates: 14 during pre-laying, 27 during peak-laying, 83 during late-laying and 80 during end-laying. The recording audit, threshold sensitivity and retained dates are reported in Supplementary Section S1.

### 2.3. RFID Record Validation, Antenna Mapping and Cleaning

Source RFID records were validated, spatially mapped and cleaned before transitions were constructed. Antenna-level RFID data can contain brief or repeated detections caused by signal interruption, overlapping antenna detection fields or rapid alternation among antennas, rather than genuine changes in functional location [27,33]. For example, a hen may reposition within a feeder or nest-box area and be detected by several antennas without leaving that functional area. RFID sequences are therefore commonly processed using duration and bout criteria that exclude very short events and merge detections separated by brief within-area gaps [27,28].

Raw antenna identifiers were matched to the flock-specific antenna-management map and grouped into the functional areas represented in the analysis. Indoor row-specific locations were assigned hierarchical identifiers of the form Pen:Row:Area. For example, P4:R1:LF denotes the lower-feeder area in Row 1 of P4. The monitored indoor areas comprised the lower feeder (LF), nest box (NB) and upper feeder (UF) in each of the two aviary rows. Locations without a row designation were assigned identifiers of the form Pen:Area and included the pop-hole and outdoor range in each housing section and the outdoor range feeder (abbreviated RF in figures) present only in P4. Detections from the inner and outer pop-hole readers were assigned to the corresponding Pop-hole category. For section-specific and pooled summaries, the pen prefix was omitted from the displayed location labels because the housing section was either fixed within the panel or accounted for during pooling. The mapping step assigned records to a common spatial resolution but did not itself merge or remove records.

Each source record was then subjected to basic temporal-validity and spatial-consistency checks. A record was provisionally valid only when: (i) both entry and exit timestamps were available; (ii) the entry timestamp corresponded to the source-file date; (iii) the RFID identifier was present in the official flock roster; (iv) a documented housing-section allocation was available for the corresponding date; (v) the antenna could be mapped to an analysed functional area; (vi) the housing section encoded by the antenna agreed with the hen’s documented allocation; and (vii) the exit time was not earlier than entry time.

Housing-section membership was obtained from the official flock-management records rather than inferred from RFID detections. For records collected up to 18 December 2016, the expected housing section was determined using each hen’s original allocation (Old Pen). For records collected from 20 December onwards, it was determined using the documented post-rearrangement allocation (New Pen). The rearrangement night of 19 December was not included among the retained recording dates. Reader–assignment mismatches and records that could not be mapped to an analysed functional area were excluded. Details of the antenna-mapping audit and reader–roster agreement are provided in Supplementary Section S2.

For each provisionally valid source record, bout duration was calculated as:

Exit time − Entry time.

Following a processing rule used previously for the same commercial RFID project [19], source bouts lasting at least 10 s were retained. In the 20-date route-sensitivity sample, increasing the minimum from 1 to 10 s, with the merge gap held at 10 s, reduced all non-looping counts by 0.16–0.39%. A separate check excluded every feeder-related endpoint, including RF; its absolute route-count changes are reported in Table S8. This sample included allocation-valid hens before final-cohort restriction and is not a full-cohort analysis of all 204 dates.

After application of the minimum-duration rule, the mapped bouts were ordered chronologically within each hen and retained calendar date. Consecutive bouts assigned to the same functional area were merged when the effective gap between them was ≤10 s. The effective gap was calculated as the entry time of the subsequent bout minus the latest exit time reached within the current same-area run. This approach allowed overlapping and chained same-area records to be combined correctly. Each merged bout retained the first entry time and the latest exit time of its contributing source records.

The 10-s merge gap combined short interruptions within the same mapped area. In the original sensitivity audit, 98.31% of merged links involved different antennas mapped to that area. The selected rule preserved all top-20 route sets, with minimum rank correlation 0.9988; at 15 s, minimum overlap was 95% (Table S3). Because feeders dominate these rankings and same-area merging leaves cross-area counts unchanged by construction, rank stability was treated as a description of sensitivity, not independent validation of the threshold.

No maximum source-bout duration threshold was imposed. Unusually long bouts were retained and flagged during quality control rather than removed using an arbitrary upper limit. Cleaning and transition construction were performed independently within each hen and retained calendar date. Sequences were restarted at the beginning of every retained date; consequently, no transition was constructed across midnight, an excluded recording date or a missing source-file date.

Same-area temporal overlaps were merged using the latest exit within the current same-area run. Different-area overlaps were retained in entry-time order and flagged rather than assigned an unobserved physical order. The final cohort contained 13 negative exit-to-next-entry cross-area gaps: five in late-laying and eight in end-laying. Timing sensitivities excluded these ambiguous pairs without creating replacement links (Supplementary Section S8.4).

### 2.4. Construction of the Final Analysis Cohort

The official flock roster contained 3125 RFID-tagged hens, of which 3118 had documented post-rearrangement assignments. Seven hens without a documented new assignment were not eligible for post-rearrangement analyses.

The final cohort comprised hens with at least one valid cleaned RFID bout on retained dates in every stage. This stage-presence rule did not require detection on every date or on a specified proportion of dates. It retained 2162 hens; 956 of the 3118 hens with a post-rearrangement assignment failed the rule. Together with seven hens lacking that assignment, 963 of the original 3125 tagged hens were excluded.

For pre-laying analyses, these hens were grouped according to their original housing sections, giving 447 hens in P1, 420 in P2, 393 in P3, 438 in P4 and 464 in P5. For peak-, late- and end-laying analyses, the same hens were grouped according to their post-rearrangement assignments, giving 382 hens in P1, 453 in P2, 440 in P3, 445 in P4 and 442 in P5. Relative to the original allocation of 625 hens per section, 178, 205, 232, 187 and 161 hens from original P1–P5, respectively, were not included in the final cohort.

Of the 2162 cohort hens, 444 received the same numbered housing-section assignment before and after rearrangement, whereas 1718 changed numbered sections. Thus, the pooled stage-level analyses followed the same individual hens throughout the four stages, but a numbered section during pre-laying did not contain the same group of hens as the corresponding numbered section after rearrangement. Comparisons among peak-, late- and end-laying followed the established post-rearrangement groups, whereas comparisons between pre-laying and subsequent stages were interpreted as housing-section-level rather than within-group longitudinal comparisons. Cohort construction, detection coverage and sensitivity to alternative inclusion and denominator rules are detailed in Supplementary Section S3.

### 2.5. Transition Construction and Rate Calculation

Transition construction used the mapped functional-area labels defined in Section 2.3. During record validation and cleaning, row-specific indoor locations retained their full identifiers in the form Pen:Row:Area such as P4:R1:LF. Row-independent locations were identified by pen and area, such as P4:RF. For transition tables and visualisations, the housing section was reported separately or fixed within each panel; therefore, the pen prefix was omitted from the displayed location labels. Indoor locations were consequently represented at the row level as R1:LF, R1:NB, R1:UF, R2:LF, R2:NB and R2:UF, together with Pop-hole, Range and RF where applicable. Pooled analyses combined corresponding mapped locations across P1–P5.

Within each retained hen-day, cleaned bouts were ordered chronologically by entry time. Each pair of adjacent bouts was used to form one directed transition. For example, the sequence A, B, C generated the transitions A→B and B→C. A cleaned detection bout comprised either one retained source event or consecutive same-area source events combined by the merging rule. Bouts were ordered separately for each hen and retained calendar date. Each detected hen-day containing *n* cleaned bouts contributed *n* − 1 loop-inclusive transitions; a hen-day containing a single bout contributed no transitions but remained in the detected hen-day denominator.

Two complementary transition representations were constructed. The loop-inclusive representation retained every adjacent pair of cleaned bouts, including A→A self-loops. For example, the sequence LF, LF, LF, NB generated two LF→LF transitions and one LF→NB transition. In the non-looping representation, only transitions between different functional areas were retained; equivalently, adjacent runs of identical area labels were collapsed before transitions were counted. The same example therefore generated one LF→NB transition.

Self-loops were treated as indicators of repeated detections in the same functional area rather than as movements between distinct areas. An A→A self-loop showed that the same functional area was recorded in two consecutive cleaned bouts, but it did not establish that the hen remained continuously in that area between the two detections. For self-loop *i*, the gap was calculated as:

$${\mathrm{Entry}}_{i+1}-{\mathrm{Exit}}_{i}$$

No upper cap was applied to these gaps. Their distribution and interpretation are described in Supplementary Section S4.

Transition rates were expressed per detected hen-day. A detected hen-day was defined as a final-cohort hen on a retained date with at least one valid cleaned RFID bout. A hen-day with a single cleaned bout contributed to the detected-hen-day denominator but contained no adjacent transition. For a transition from location (*i*) to location (*j*), within housing section (*p*) and production stage (*s*), the rate was calculated as:

$$\frac{N_{i\to j,p,s}}{H_{p,s}^{\det}},$$

where $\left(N_{i\to j,p,s}\right)$ is the observed transition count and $\left(H_{p,s}^{\det}\right)$ is the number of detected hen-days in the corresponding housing section and stage.

For pooled stage-level summaries, transition counts and detected-hen-day denominators were summed across the five housing sections before the rate was calculated:

$$\frac{\sum_{p=1}^{5}N_{i\to j,p,s}}{\sum_{p=1}^{5}H_{p,s}^{\det}}$$

Pooled rates were calculated from summed counts and summed detected hen-days, rather than by averaging the five section rates. Eligible hen-days comprised all cohort hens multiplied by retained dates, including dates without a valid detection. Both denominators were reported. Detected-hen-day rates describe recorded transitions conditional on at least one valid bout that day; they do not estimate activity on undetected days or correct missed detections within a detected day (Supplementary Section S3).

Within each housing section and production stage, loop-inclusive, self-loop and non-looping transition rates were calculated using the same detected hen-day denominator. Consequently, the loop-inclusive rate equalled the sum of the self-loop and non-looping rates. The same relationship held for pooled rates calculated from transition counts and detected hen-days summed across housing sections.

### 2.6. Sensitivity Analyses and Descriptive Variability

The primary estimand was the recorded transition rate in the retained cohort and dates. We reported the five observed section rates and the pooled rate, together with medians and quartiles of individual hen-stage rates. Each individual rate used that hen’s transition count divided by its detected dates in the stage. Quartiles describe observed heterogeneity and are not confidence intervals. No hypothesis tests or model-based population-effect estimates were calculated; the five sections were not treated as independent flock or treatment replicates.

Four complementary checks assessed sensitivity to observation and selection choices. First, eligible-hen-day rates were compared with detected-hen-day rates. Second, rates were recomputed in the 1480 hens detected on at least 75% of retained dates in every stage. Third, the retained hens were compared descriptively with the 956 excluded hens having documented assignments, using available early weights and original pop-hole detection summaries. Seven hens without assignments were kept separate. Fourth, original non-looping pairs were restricted either to exclude Range/RF endpoints or to have both endpoints among the six indoor feeder/nest-box nodes. Events were not deleted and reconnected; original full-network detected hen-days remained the denominator. These restrictions do not equalise antenna uptime or detection probability.

Timing checks reconstructed the primary counts from the cleaned daily bouts. Existing within-day pairs were retained only when exit-to-next-entry gaps were nonnegative and no longer than 15, 60 or 240 min, with an additional scenario having no upper gap limit. These were sensitivity thresholds, not validated boundaries for physical movement. A separate check added eligible non-looping pairs between consecutive retained calendar dates in the same stage and section, with the same four limits applied only to added pairs. Existing within-day links and denominators were held fixed. Missing or excluded dates and stage boundaries were not bridged. Same-area boundary candidates were counted separately; continuous overnight bouts and loop counts were not rebuilt (Tables S10 and S11).

### 2.7. Transition Visualisation

**Figure 2:**
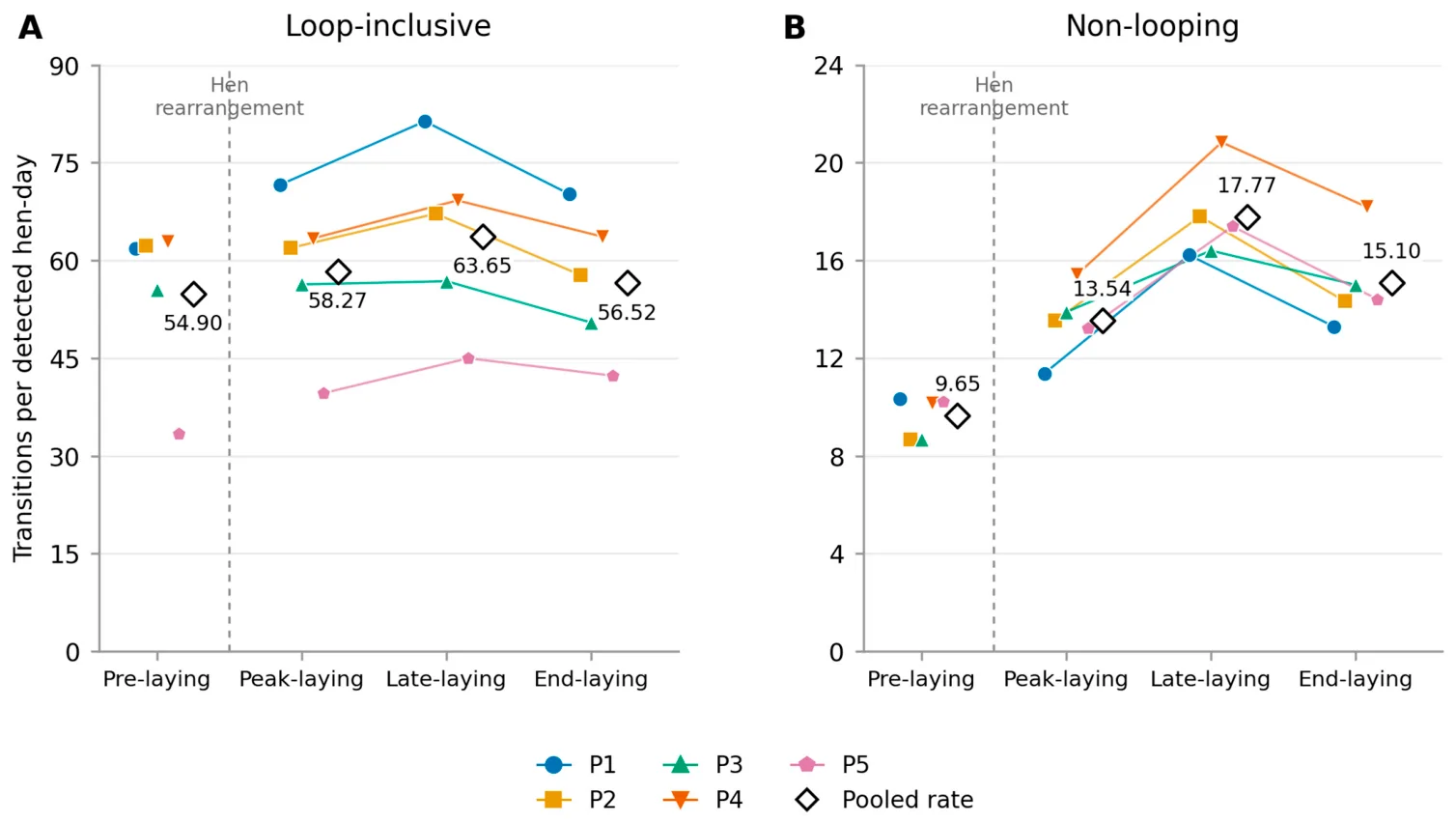
Recorded transition rates across production stages. (**A**) Loop-inclusive and (**B**) non-looping transitions. Coloured points show the five physical housing sections. Diamonds show pooled rates from summed counts divided by summed detected hen-days. Lines connect section values only after rearrangement; numbered sections changed composition between pre- and peak-laying. These sections belong to one flock and are not independent flock replicates. The points and pooled values are descriptive; no confidence intervals are displayed.

**Figure 3:**
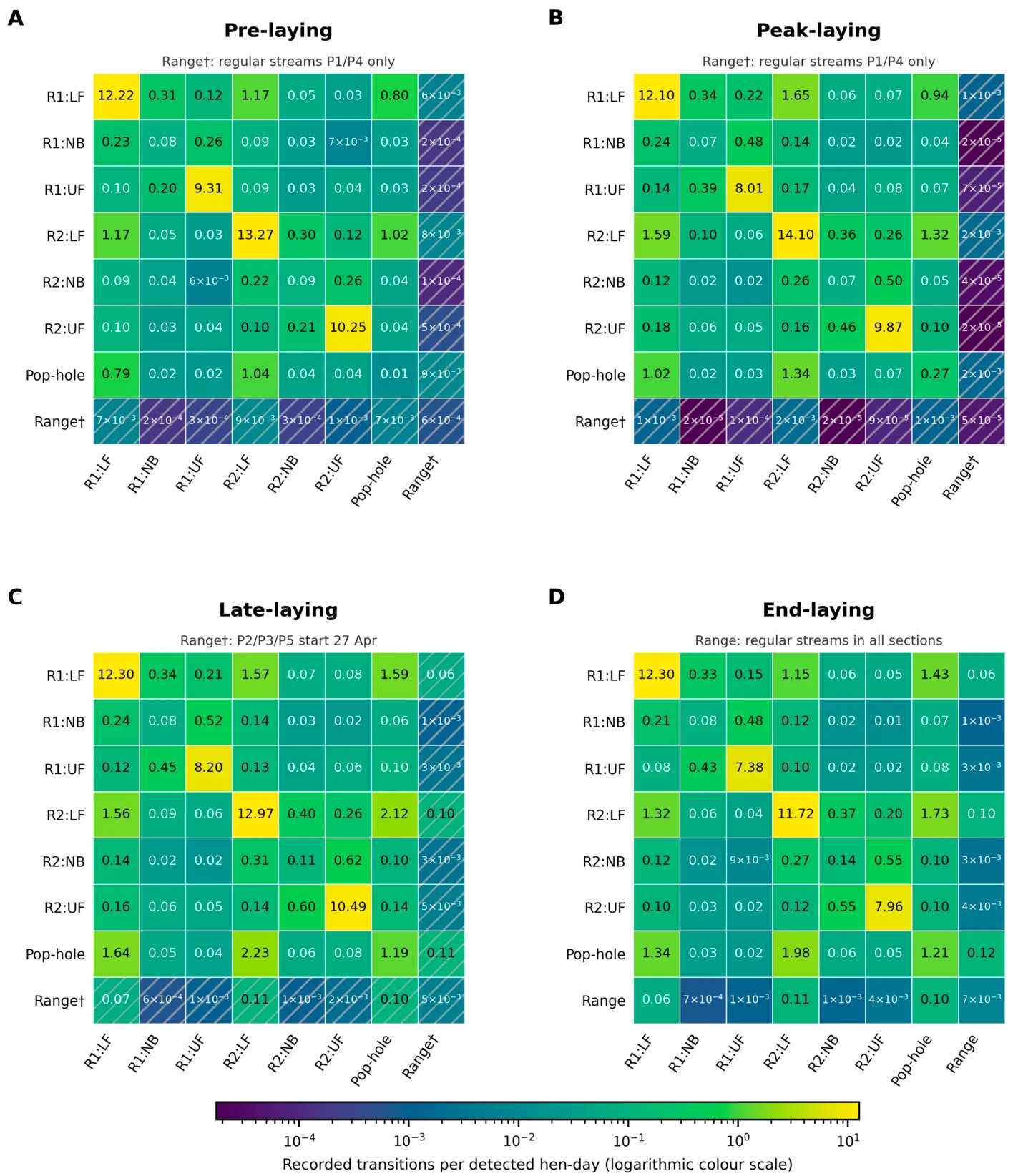
Pooled transition heatmaps for (**A**) pre-, (**B**) peak-, (**C**) late- and (**D**) end-laying. Cells show transitions per detected hen-day; rows are origins and columns destinations. Diagonals are self-loops. All panels share a logarithmic colour scale. Hatching, dagger marks and panel notes flag unequal Range coverage: regular streams were available in P1/P4 earlier and began in P2/P3/P5 on 27 April 2017. RF routes are omitted here and shown separately in Figure 4. LF, lower feeder; NB, nest box; UF, upper feeder; R1/R2, aviary rows. NR denotes no regular recording stream. Small positive rates use scientific notation.

**Figure 4:**
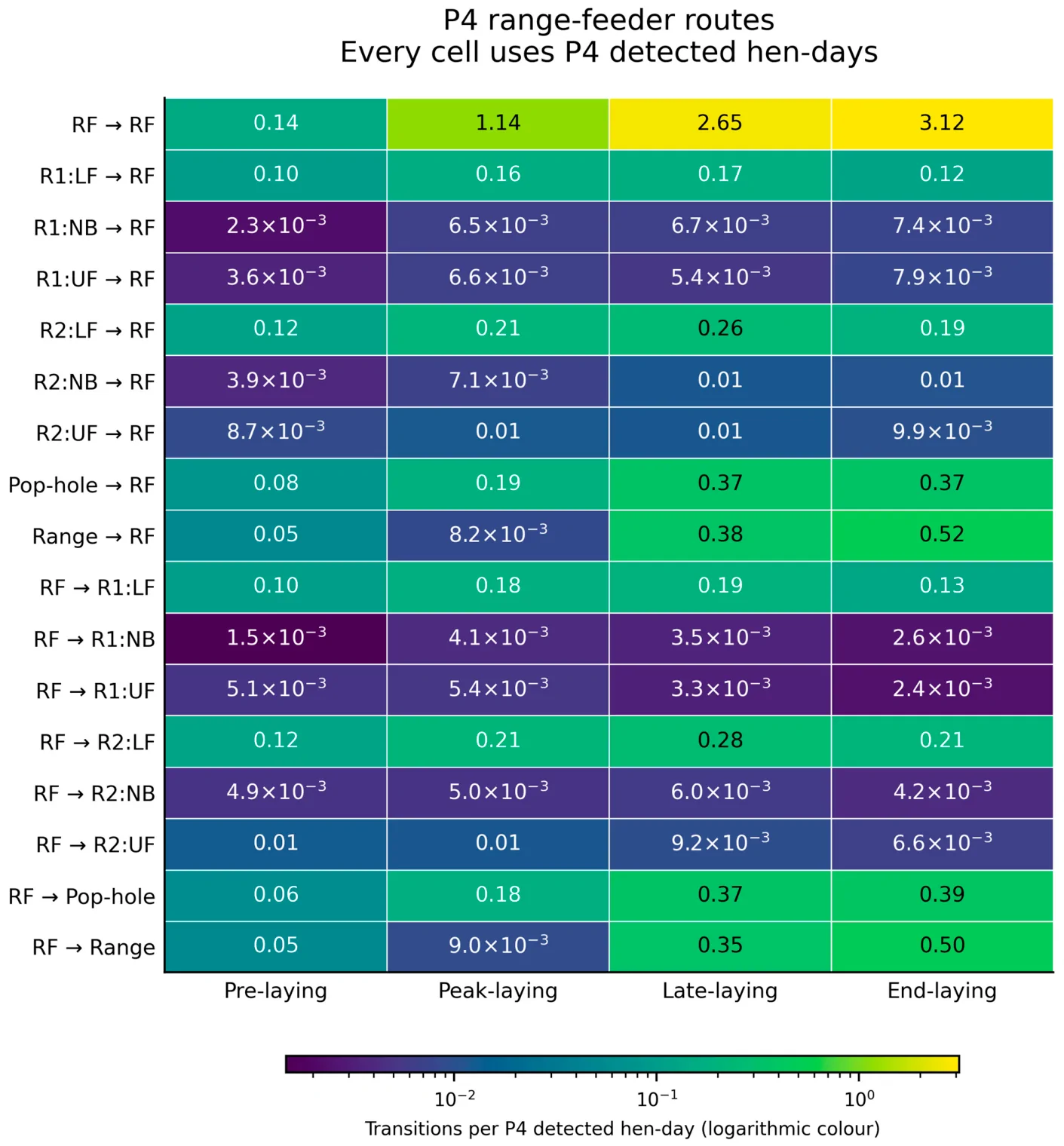
All 17 directed RF-linked routes in P4 across stages, including RF→RF. Rates use P4 detected hen-days throughout. The separate logarithmic colour scale summarises these local rates; exact annotations provide the comparison. RF, outdoor range feeder; other abbreviations follow Figure 3. The figure describes one section and does not estimate a resource effect.

The main presentation comprised section points and pooled rates (Figure 2), four stage heatmaps using the same row/column order and colour scale (Figure 3), and a separate P4 range-feeder comparison using P4 denominators (Figure 4). Main pooled heatmaps omit RF-linked cells; their remaining counts and five-section denominators are unchanged. Complete section-level heatmaps are grouped into four stage panels per section (Figures S1–S5). All heatmaps retain self-loops, and rows and columns denote origins and destinations.

Positive heatmap rates use a base-10 logarithmic colour scale, capped at the 98th percentile of positive rates across the four pooled and 20 section matrices. This affects colour intensity only. Rates are annotated; selected route counts and denominators are reported in the Supplementary tables and interactive diagrams. Grey zero cells denote no recorded transition in a represented location pair. Hatching and NR (no regular recording stream) identify cells without a regular Range stream, and dagger symbols with on-panel notes identify partial coverage. RF is structurally absent outside P4 and is omitted from other section matrices.

Sankey diagrams are retained as an offline Supplementary interactive file. Locations appear in origin and destination columns, and ribbon widths represent recorded transition rates within each selection. The display retains routes at or above the 80th percentile of positive counts after selecting the section/stage and loop representation. Pooled views omit RF; its rates are available under P4. Widths are independently scaled, so comparisons use numerical rates. A fixed set of the same 15 prominent non-looping routes across the four original pooled stage comparisons is shown with rate labels in Figure S6. Omission from a filtered diagram is not a measured zero.

### 2.8. Software and Ethical Approval

Primary RFID processing used Python 3.13.9 after extraction from a PostgreSQL database (https://www.postgresql.org/; server version not documented in the available records). Additional table checks and publication-figure assembly used Python 3.12.14, pandas 2.2.3, NumPy 2.3.5 and Matplotlib 3.10.8. The Supplementary interactive diagrams were provided as self-contained Hypertext Markup Language (HTML) with Scalable Vector Graphics (SVG) and JavaScript controls.

The original animal study was approved by the University of New England Animal Ethics Committee (approval number AEC 16-087). The present work is a secondary analysis of those records and involved no additional animal procedures. We report the applicable design, animal, cohort, outcome and analysis details with reference to the ARRIVE 2.0 reporting guidelines [34]. The original animal ethics protocol was not separately registered before data collection. Information on adverse events, individual-hen humane endpoints and the frequency of welfare checks could not be verified from the records used for this secondary analysis.

## 3. Results

All comparisons below describe the retained records. Production stage, age, calendar time, lighting, range-access history and recording coverage changed together, and their contributions were not separately estimated.

### 3.1. Overall Transition Activity

Following cleaning and restriction to the final four-stage cohort, the dataset contained 24,155,194 cleaned detection bouts across 398,488 detected hen-days. These daily sequences yielded 23,756,706 transitions, comprising 17,549,442 self-loops (73.9%) and 6,207,264 non-looping transitions (26.1%).

Pooled non-looping rates were 9.65, 13.54, 17.77 and 15.10 transitions per detected hen-day across the four stages (Table 1). Self-loop rates were 45.25, 44.72, 45.88 and 41.43. Thus, the increase in loop-inclusive rates through late-laying was mainly associated with cross-area links. Figure 2 shows the five section values and the pooled estimate, with section lines restricted to the post-rearrangement stages.

Individual non-looping rates also varied within each stage. Median [Q1, Q3] rates were 9.93 [6.14, 12.93], 13.56 [11.07, 16.04], 17.31 [14.16, 21.02] and 14.90 [11.96, 18.00] transitions per detected hen-day. These summaries describe the 2162 hen-stage rates, whereas pooled rates weight hens by their detected days (Table S9).

### 3.2. Principal Cross-Area Connections

Lower feeders participated in the highest-ranked non-looping route in every section–stage combination (Table S6). Cross-row lower-feeder routes were most prominent in peak-laying: pooled R1:LF→R2:LF and reverse rates were 1.65 and 1.59, compared with 1.17 in both directions during pre-laying. Later, pop-hole–lower-feeder routes were strongest. Pop-hole→R2:LF and its reverse reached 2.23 and 2.12 during late-laying and 1.98 and 1.73 during end-laying (Figure 3).

Nest-box-linked routes accounted for 26.37%, 27.87%, 24.69% and 26.06% of pooled non-looping transitions across stages. They mainly connected nest boxes with feeders, commonly within the same row. The modest peak-stage maximum concerns the recorded share of these links and does not identify individual laying events. Figure S6 compares the same 15 prominent routes across stages using numerical rates.

Feeder self-loops occupied the four highest positions in all 20 section–stage rankings. The four indoor feeders contributed 99.53%, 98.55%, 95.83% and 94.99% of self-loops across stages (Table S5). These repeated detections dominate total counts but do not identify feeding or a departure and return. Their gap distributions are provided in Section S4 rather than interpreted as residence durations.

### 3.3. Local Range-Feeder Pattern in P4

P4-specific RF→RF rates were 0.14, 1.14, 2.65 and 3.12 transitions per detected hen-day across stages. Range↔RF and Pop-hole↔RF links became more prominent later (Figure 4). All RF-linked rates shown here use P4 detected hen-days. They describe one equipped section; there is no replicated contrast estimating a feeder, diet or prior range-use effect.

### 3.4. Detection Coverage and Robustness

Detection coverage declined from 99.21% to 84.19% across the four stages. Using eligible rather than detected hen-days gave non-looping rates of 9.57, 13.21, 16.43 and 12.71; the denominator changed the end-laying rate most strongly (Table 2). The higher-coverage 1480-hen subset gave 9.37, 13.34, 17.63 and 14.95. These checks retained the stage ordering but do not quantify missed transitions within a detected day.

Restricting existing links to the six indoor feeder/nest-box locations gave rates of 5.52, 8.26, 8.49 and 7.02 per original detected hen-day. The peak-to-late increase was 2.7%, compared with 31.2% for all non-looping links. Excluding only Range and RF links gave 9.45, 13.29, 16.70 and 14.03 (Table S12). The magnitude of the contrast therefore depended strongly on the locations included. Restriction to common location labels does not establish equal antenna operation or detection sensitivity.

The retained and excluded assigned hens had the same median initial weight, 1.364 kg, but differed in their original early pop-hole detection summaries: median cumulative recorded time was 89.08 min in retained hens and 23.96 min in excluded hens. Missing 22-week weights were also more frequent among excluded hens (313/956 versus 60/2162). These comparisons indicate selection differences in recorded histories; they do not identify why individual hens were excluded (Table S13).

In the 20-date cleaning sample, the strict non-feeder subset changed from 2698 to 2701, 2143 to 2143, 5516 to 5529 and 5544 to 5555 links when the minimum bout duration increased from 1 to 10 s at a fixed 10-s merge gap. Sum-of-absolute route changes were 0.11–0.24% of baseline counts (Table S8). The small increases in some subset totals arise because excluding an intervening short bout can make two other locations adjacent.

Applying a 15-min upper gap limit reduced non-looping counts by 45.57–58.27%; a 60-min limit reduced them by 6.57–13.58%, and a 240-min limit by 0.04–0.10%. Stage ordering remained unchanged under every limit (Table S10). Adding nonnegative cross-midnight links between consecutive eligible retained dates increased stage counts by 0.53–0.89% without an upper limit, and by at most 0.025% with a 240-min limit (Table S11). These are separate sensitivities: boundary additions did not remove existing long-gap within-day pairs. The 13 negative-gap cross-area pairs represented 0.00021% of primary non-looping transitions.

## 4. Discussion

### 4.1. Contribution of the Transition Representation

The sequence representation adds information that duration summaries alone do not retain: which monitored areas were successive, how strongly particular directed pairs contributed to the record, and how repeated same-area detection influenced total transition activity. It provides a descriptive account of cross-area adjacency with an explicit separation of a large repeated-detection component. This complements duration-based features from the same programme [19] and the movement-monitoring examples discussed by Ruhnke et al. [30].

Lower feeders were successive to many of the strongest links, whereas the relative prominence of feeder–pop-hole connections increased later in the record. Separating self-loops showed that the rise in loop-inclusive activity through late-laying was associated mainly with non-looping transitions. However, the much smaller peak-to-late increase among the six indoor locations is equally important: the apparent magnitude of change depends on the monitored part of the network.

### 4.2. Temporal Definitions and Repeated Detection

RFID fragmentation and intermittent detection make bout definitions consequential [27,28,33,35,36]. The minimum 10-s bout criterion has a precedent in Welch et al. [19]; the same-area merge rule is an additional operational definition. The non-feeder check extends the sensitivity assessment beyond feeder-dominated rankings. It supports the limited effect of the minimum-duration choice on the sampled cross-area routes but is not independent behavioural validation. Invariance of non-looping counts under same-area merging is an algebraic consequence of merging identical adjacent labels, not evidence that a gap threshold is biologically correct.

Because approximately 95–99.5% of self-loops occurred at indoor feeders, tag repositioning and fragmentation within antenna fields are plausible major contributors. Some self-loops could also span unobserved departures or use of the unmonitored litter area. The available records cannot separate those causes. Self-loop rates and gaps are consequently operational summaries of repeated detection, with detailed distributions in the Supplement. Electronic-nest laying durations [37] concern a different quantity and should not be inferred from these gaps.

Calendar-day segmentation excluded midnight-spanning links. The boundary check indicates a small count effect for non-looping adjacency between consecutive retained dates. It does not reconstruct continuous same-area bouts or quantify overnight perching: the 287,083 same-area boundary candidates include 222 gaps of at most 10 s, and their treatment would require reapplying merging to continuous sequences. Nor does the check recover links across excluded dates. Maintaining those breaks avoids inventing sequences through unobserved intervals but may preferentially omit long interruptions. The reported gap-cap scenarios expose that dependence without defining a validated travel-time cutoff.

### 4.3. Stage Patterns and Observation Coverage

The retained-date stages followed the sequence from early to later production, but also differed in age, season, lighting and range-access history. Pre-laying followed progressive range opening and preceded rearrangement. Interpretation of early activity should therefore consider acclimatisation and access history [9], while the later feeder–pop-hole prominence describes recorded use of the indoor–outdoor interface. Nest-box-linked transitions had a modest peak-stage maximum, but individual oviposition was not measured.

Earlier studies help place feeder-associated records in context. Duration-based analyses linked lower-tier and range use [19,30,31], and Kolakshyapati et al. [38] reported associations with initial body weight and exploration-related measures. Age-related spatial shifts [39] and strain differences in resource use [40] also show that occupancy depends on animal and housing characteristics. These results provide context rather than validation of a specific route or explanation for a stage contrast in the present flock.

Neither the detected-day denominator nor the higher-coverage cohort corrects antenna-specific sensitivity, tag orientation or missing intermediate locations. Restriction to shared indoor labels retained the broad stage ordering but substantially reduced one headline contrast. Thus, recorded stage differences should not be presented as if monitoring exposure were equivalent. Matching equipment, operation periods and observation opportunity across stages would give a stronger comparison; the present summaries quantify sensitivity to available restrictions, rather than estimating the separate contributions of hardware and behaviour.

### 4.4. Range and Housing-Section Contrasts

Regular Range streams in P2, P3 and P5 began on 27 April 2017, so early pooled Range values mainly represent P1 and P4. The revised figures flag this directly and distinguish absent regular coverage from measured zero counts. RF routes are shown separately with P4 denominators. This avoids diluting a local resource with five-section exposure, although its unreplicated association with P4 remains. Outdoor resources, cover, weather and experience can influence range use [41,42,43,44,45,46]; those factors were not separately adjusted for here.

Earlier pop-hole monitoring studies provide context for describing activity around this interface [47]. Merging inner and outer pop-hole readers in the present analysis preserved a common area node but discarded the side order needed to classify an inward or outward crossing. Directional reconstruction is potentially feasible from original reader-level sequences during periods with confirmed paired-reader mapping and operation. The cleaned bouts retain a first-reader summary rather than the complete reader-side sequence, and Inner_Pophole labels first appear in the audit on 9 February 2017. That first label date cannot establish when equipment was installed or consistently paired. A directional sub-analysis therefore requires a separate raw-reader reconstruction and validation.

Post-rearrangement range-use groups and management conditions each occupied a particular section. Earlier findings about health or range-use groups [32,48,49] therefore cannot be used to assign causes to present section differences. In particular, the P5 self-loop pattern was also present before rearrangement, when occupants differed. This supports caution about a diet-only or phenotype-only explanation, while leaving detection and physical section characteristics unresolved.

### 4.5. Limits of Inference and Future Work

The study concerns selected observed records from one commercial flock. The cohort excludes hens without valid records in every stage, and retained versus excluded hens differed in early recorded pop-hole use and later weight availability. The estimates should not be generalised to all originally tagged hens or used to infer causes of mortality, tag loss or exclusion. Initial observed weight similarity does not demonstrate absence of selection bias.

Figure 2 and the individual-rate quartiles make the observed spread visible. They do not supply uncertainty intervals for a causal stage or treatment effect. No such effect was estimated, and a large number of detections does not provide independent replication of a flock, season, diet or resource condition. Statistical count models could examine conditional hen-level associations but would not by themselves remove the design confounding or establish sensor validity. The present standalone question is the structure and observation-sensitivity of recorded transitions; it does not depend on results promised for a subsequent model.

Validation against synchronised video or direct observation would be needed to estimate missed or erroneous detections and distinguish behavioural use from fragmentation. Existing validation work demonstrates that performance can differ markedly with spatial resolution [18]. Replication across flocks and a stable, documented antenna configuration are needed before these descriptive route patterns can support broader biological or management conclusions.

## 5. Conclusions

Separating same-area self-loops from cross-area links provided complementary summaries of the RFID records in this 2162-hen cohort. Repeated feeder detections dominated counts, while lower feeders participated in the strongest cross-area links and feeder–pop-hole connections were more prominent later in the retained period. This sequence information complements duration-based summaries.

The size of the stage contrasts depended on observation choices, particularly the locations included, and recording coverage changed over time. The findings therefore describe this retained cohort and monitoring configuration. They do not establish separate age, season, diet, phenotype or feeder effects. The combination of explicit definitions, accessible aggregate summaries and sensitivity checks provides a basis for future replicated and behaviourally validated analyses.

## Figures and Tables

**Table 1:** Pooled recorded transition rates across production stages. Counts and detected hen-days were summed across P1–P5 before division. Loop-inclusive rates equal self-loop plus non-looping rates before rounding.

Production Stage	Loop-Inclusive Rate	Self-Loop Rate	Non-Looping Rate
Pre-laying	54.90	45.25	9.65
Peak-laying	58.27	44.72	13.54
Late-laying	63.65	45.88	17.77
End-laying	56.52	41.43	15.10

**Table 2:** Main detection and observation-sensitivity summaries. All rates are non-looping transitions per hen-day. Eligible rates include all retained cohort hen-days; the other columns use detected hen-days. Indoor rates retain existing links with both endpoints among the six indoor nodes, using original full-network detected days. The higher-coverage subset contains 1480 hens detected on at least 75% of retained dates in every stage.

Stage	Detection Coverage	Primary Rate	Eligible-Day Rate	Indoor-Link Rate	Higher-Coverage Rate
Pre-laying	99.21%	9.65	9.57	5.52	9.37
Peak-laying	97.56%	13.54	13.21	8.26	13.34
Late-laying	92.45%	17.77	16.43	8.49	17.63
End-laying	84.19%	15.10	12.71	7.02	14.95

## Data Availability

Summary results are provided in the manuscript, Supplementary Materials and accompanying interactive transition diagrams. The underlying individual RFID records are not publicly available because of confidentiality restrictions. Requests for access may be directed to the corresponding author and are subject to the applicable confidentiality requirements and permission from the data owner.

## Supplementary Materials

The following supporting information can be downloaded at: https://www.mdpi.com/article/10.3390/ani16213533/s1. Sections S1–S7 provide recording coverage, cleaning sensitivity, cohort and denominator definitions, self-loop gaps, section-level top transitions, consolidated section heatmaps and interactive transition diagrams. Section S8 reports age reconciliation and the additional cohort, location and timing checks. The supplementary document is accompanied by Supplementary_Interactive_Transitions.zip, which contains a self-contained HTML file with interactive transition diagrams and selected aggregate counts and rates.
