# Supplementary material for: Movement Patterns of Laying Hens Across Production Stages in a Commercial Aviary System
Source: Animals (Basel). 2026 Nov 9;16(21):3533. doi: 10.3390/ani16213533 (PMC13761489; doi:10.3390/ani16213533)
Supplement: Supplementary file 1 [file animals-16-03533-s001.zip › animals-4623964_Supplement_Proofread.pdf]

Supplementary Materials

S1. Recording Coverage and Retained-Date Selection

Raw radio-frequency identification (RFID) data were supplied as separate files for each available recording date. Because the recording system was intended to operate continuously but some dates were absent or incompletely recorded, temporal coverage was assessed before RFID cleaning and transition construction. The four production-stage windows contained 342 calendar dates in total. Source files were available for 280 dates, while 62 dates had no corresponding file and were therefore unavailable for analysis. Database timestamps were analysed as stored, without applying an additional clock adjustment.

Recording coverage was assessed separately for each of the five housing sections (P1–P5). To be retained, a date had to satisfy all of the following conditions: (i) its allocation phase was compatible with the production-stage analysis; (ii) all five housing sections contained RFID records; (iii) the interval between the first and last RFID entry timestamp was at least 20 h in every section; (iv) every hourly interval in the fixed audit window, from 04:00 to 19:59, contained at least one RFID record in every section; (v) the number of records in each section was at least 75% of the median daily record count for the corresponding production stage and section; and (vi) the number of active antennas in each section was at least 80% of the median number of active antennas for the corresponding production stage and section.

The ≥20-h criterion was applied to the overall recording span within each housing section. It was not a requirement that every individual hen be detected for 20 h. Similarly, the fixed-hour criterion assessed section-wide operation of the recording system rather than individual hen activity. Requiring all five sections to pass the same criteria ensured that the analyses used matched calendar dates across P1–P5. If any section failed a criterion, that date was excluded for all sections.

The allocation-phase criterion was included because hens were rearranged among housing sections on the night of 19 December 2016. Only dates up to 18 December were therefore eligible for the pre-laying analysis. The rearrangement date was excluded, and post-rearrangement dates within the nominal pre-laying window were not combined with the pre-rearrangement records. All peak-, late-, and end-laying dates occurred after rearrangement.

A sensitivity analysis was also conducted for the minimum recording-span requirement. Thresholds of ≥18, ≥20 and ≥22 h were compared while all other primary selection criteria were held constant, including the 75% record-volume requirement. These thresholds retained 205, 204 and 203 dates, respectively. Lowering the threshold from 20 to 18 h added only one late-laying date, 29 June 2017, on which recording ended at approximately 19:32 in all five housing sections and no records were present during the hourly intervals from 20:00 to 23:59. Increasing the threshold from 20 to 22 h excluded only one otherwise eligible pre-laying date, 15 December 2016, on which recording began at approximately 02:38 in all five sections but the remaining coverage requirements were satisfied. The ≥20-h criterion was retained because it excluded the substantial end-of-day recording loss admitted by the 18-h threshold while avoiding the additional loss of an otherwise well-covered pre-laying date under the 22-h threshold. The limited change in retained-date counts also indicated that date selection was insensitive to reasonable variation in the recording-span criterion.

Using the selected ≥20-h recording-span criterion and the 75% record-volume criterion, together with the remaining coverage requirements, 204 dates were retained: 14 during pre-laying, 27 during peak-laying, 83 during late-laying and 80 during end-laying (Table S1). These dates represented 72.9% of the 280 dates for which source files were available. All dates satisfying this rule also passed the active-antenna requirement; therefore, the antenna criterion did not exclude any additional dates after the other requirements had been applied.

Sensitivity analyses were conducted using stricter record-volume thresholds of 80% and 85%, while retaining the same temporal-coverage, fixed-hour, allocation-phase, and active-antenna requirements. Increasing the threshold to 80% reduced the number of retained dates from 204 to 192, whereas the 85% threshold retained 172 dates. Most of the additional exclusions occurred during late- and end-laying. The 75% criterion was selected for the primary analysis because it excluded dates with substantially reduced recording activity while avoiding unnecessary loss of otherwise well-covered dates, particularly during the later production stages.

Table S1. Recording-date availability and sensitivity of retained-date selection across production stages. Available source dates are calendar dates for which a raw daily RFID file was identified within the predefined production-stage window. Missing dates had no corresponding source file. Retained-date counts were calculated using record-volume thresholds of 75%, 80%, or 85% of the median daily count for the corresponding production stage and housing section. Under every threshold, dates were also required to be allocation-phase compatible and to meet the ≥20-h recording-span, complete fixed-hour coverage, and ≥80% active-antenna criteria in all five housing sections. The 75% threshold was used in the primary analysis.

Panel A: ≥18-, ≥20- and ≥22-h recording-span thresholds, with record volume fixed at 75%.

Production stage	≥18 h	≥20 h	≥22 h
**Pre-laying**	14	14	13
**Peak-laying**	27	27	27
**Late-laying**	84	83	83
**End-laying**	80	80	80
**Total**	**205**	**204**	**203**

Panel B: 75%, 80% and 85% record-volume thresholds, with recording span fixed at 20 h.

Production stage	Calendar dates in stage window	Available source dates	Missing source dates	Retained: 75% rule	Retained: 80% rule	Retained: 85% rule
**Pre-laying**	31	20	11	14	14	13
**Peak-laying**	64	33	31	27	27	26
**Late-laying**	143	131	12	83	81	70
**End-laying**	104	96	8	80	70	63
**Total**	**342**	**280**	**62**	**204**	**192**	**172**

S2. RFID Record Validation and Cleaning-Pipeline Sensitivity Analyses

S2.1. Source-Record Interpretation and Initial Validity Checks

Each row of the source data represented an antenna-level tag event, with the first valid read recorded as the entry time and the last valid read recorded as the exit time. Intermediate reads had already been filtered by the RFID software. The duration of each source bout was therefore calculated as:

$$\text{Source-bout duration} = \text{Exit} - \text{Entry}.$$

The two additional numeric fields in the headerless source files were audited rather than assumed to represent bout duration. The first field increased by one in 99.27% of comparable within-hen records, while the second equalled the entry-to-next-entry interval in 99.50% of comparable records. Neither field was therefore used as the bout-duration measure.

Before evaluating timing thresholds, a source bout was considered valid only when: (i) both entry and exit timestamps were available; (ii) the entry timestamp corresponded to the source-file date; (iii) the RFID identifier was present in the official flock roster; (iv) the hen had a documented housing-section allocation for the relevant pre- or post-rearrangement period; (v) the antenna could be mapped to an analysed functional area; (vi) the housing section encoded by the antenna agreed with the hen's documented allocation; and (vii) exit time was not earlier than entry time. Sensitivity analyses were performed on all source bouts satisfying these conditions on the 204 retained recording dates, before construction of the four-stage cohort.

S2.2. Antenna Mapping and Reader–Roster Housing-Section Agreement

Each antenna identifier was mapped to its housing section and functional area before the timing-based cleaning rules were applied. The housing section derived from the antenna was then compared with the hen's documented allocation for the corresponding date. The expected section was defined using Old Pen for records collected up to 18 December 2016 and New Pen for records collected from 20 December onwards. Records from the rearrangement date, 19 December, were excluded.

Across the complete raw-data audit, 6,905,951 records showed disagreement between the reader-derived section and the documented allocation, representing 9.20% of records for which both a reader-derived and roster-derived section were available. The mismatch rate was 5.62% during pre-laying, 5.41% during peak-laying, 8.31% during late-laying and 12.66% during end-laying.

Among the deliberately sampled mismatch examples, 97.2% were recorded at lower- or upper-feeder antennas. The most frequently observed mismatches were between neighbouring housing sections, including P5⟷P4, P3⟷P2, P4⟷P3 and P2⟷P1. This spatial pattern was consistent with cross-boundary RFID detection, particularly around feeder readers near section boundaries. However, because these records were deliberately sampled as mismatch examples, their antenna distribution should not be interpreted as an estimate of mismatch prevalence across the complete dataset.

The flock-management records confirmed that hens were rearranged only once and that no planned transfers or additions occurred after rearrangement. The official allocation records were therefore treated as the authoritative source of housing-section membership. A record was retained only when the reader-derived section agreed with Old Pen before rearrangement or New Pen after rearrangement. Reader–assignment mismatches were excluded before the duration and same-area merging rules were evaluated.

A further 578 records could not be mapped to an analysed functional area; all occurred on 20 December 2016. These represented fewer than 0.001% of the audited source records and were excluded because their reader-derived housing section and functional area could not be validated. Seven hens lacked a documented post-rearrangement assignment; their post-rearrangement records could not be validated and these hens were consequently ineligible for the final four-stage cohort, as described in Section S3.

S2.3. Minimum Source-Bout Duration

Across the complete raw-data audit, 32,113,138 of 75,132,004 source records (42.74%) had identical entry and exit timestamps. This was expected when the first and last valid reads occurred within the same one-second timestamp interval, but very short detections could also arise when stray radiofrequency signals briefly energised a tag outside the intended detection area. Minimum-duration thresholds of ≥1, ≥3, ≥5, and ≥10 s were therefore compared.

Across the 204 retained dates, 54,649,003 source bouts passed the initial validity checks. Requiring a duration of at least 1 s retained 57.42% of these bouts, showing that most of the reduction resulted from removing zero-second detections (Table S2). Increasing the threshold from ≥1 to ≥10 s removed an additional 1.66–2.36 percentage points of the valid source bouts across stages. The corresponding overall reduction was 1.97 percentage points.

In the supplied 20-date joint sensitivity sample, increasing the minimum duration from 1 to 10 s at a fixed 10-s merge gap reduced pooled non-looping counts by 0.387%, 0.297%, 0.157% and 0.237% across stages (range 0.16–0.39%). These route checks concern allocation-valid hens before final-cohort restriction and should not be described as full-cohort results on all dates. Independent non-feeder and nest-linked checks are reported in Section S8.2 and Table S8. A bout lasting exactly 10 s was retained. The minimum-duration criterion has published precedent in the same project; neither threshold was validated here against observed behaviour.

Table S2. Sensitivity of source-bout retention to the minimum-duration threshold. Counts include all valid roster-, allocation-, antenna-, and date-matched source bouts on the 204 retained recording dates, before same-area merging and four-stage cohort construction. Percentages are calculated relative to the number of valid source bouts within each production stage.

Production stage	Valid source bouts	Retained at ≥1 s, n (%)	Retained at ≥3 s, n (%)	Retained at ≥5 s, n (%)	Retained at ≥10 s, n (%)
**Pre-laying**	4,239,171	2,582,525 (60.92%)	2,544,122 (60.01%)	2,518,502 (59.41%)	2,482,627 (58.56%)
**Peak-laying**	7,468,160	4,648,344 (62.24%)	4,600,250 (61.60%)	4,568,522 (61.17%)	4,524,532 (60.58%)
**Late-laying**	23,868,519	13,807,199 (57.85%)	13,640,400 (57.15%)	13,529,249 (56.68%)	13,369,040 (56.01%)
**End-laying**	19,073,153	10,341,378 (54.22%)	10,180,428 (53.38%)	10,076,560 (52.83%)	9,927,692 (52.05%)
**Total**	**54,649,003**	**31,379,446 (57.42%)**	**30,965,200 (56.66%)**	**30,692,833 (56.16%)**	**30,303,891 (55.45%)**

S2.4. Same-Area Merge-Gap Threshold

After applying the ≥10-s minimum duration, bouts were ordered separately for each hen and calendar date. Consecutive bouts assigned to the same functional area were considered candidates for merging. To handle overlapping and chained bouts correctly, the effective same-area gap was calculated as the entry time of the subsequent bout minus the latest exit time already reached within the current same-area run. Bouts were merged when this effective gap was less than or equal to the selected threshold. The merged bout retained the first entry time and the latest exit time of its contributing source bouts.

Candidate merge thresholds of 0, 5, 10, 15, 20, 25, 30, 45, and 60 s were evaluated. The numbers and proportions of same-area links merged were calculated across all 204 retained dates. Route stability was assessed using five approximately evenly distributed retained dates from each stage, giving a 20-date sensitivity sample. For each threshold, route rankings were compared with the ≥10-s minimum-duration dataset without same-area merging.

A threshold of ≤10 s merged 2,714,806 of 22,817,535 adjacent same-area links (11.90%). Of the merged links, 98.31% involved different physical antennas mapped to the same functional area. In contrast, only 1.82% of all links involving repeated detections at the same individual antenna were merged. The composition of the gap distribution also changed immediately beyond 10 s: same-antenna links represented only 2.55% of links in the

5–10-s interval but 17.45% of links in the 10–15-s interval. This change suggests that extending the threshold beyond 10 s would increasingly combine repeated detections from the same antenna, rather than primarily resolving rapid switching among antennas covering the same functional area.

The 10-s threshold preserved the top-20 route set in every assessed section-stage comparison, with minimum Spearman rank correlation 0.9988 (Table S3). Minimum overlap declined to 95% at 15 s. These feeder-dominated rankings describe stability under processing choices rather than independent threshold validation. Same-area merging leaves cross-area counts unchanged by construction. The selected 10-s definition is operational; its biological validity requires separate observations.

Table S3. Sensitivity of same-area merging to the effective exit-to-next-entry gap threshold. Same-area merge counts were calculated from 22,817,535 adjacent same-area links across all 204 retained dates after applying the ≥10-s minimum source-bout duration. Route-rank correlations and top-20 overlap values were calculated from the 20-date sensitivity sample and are the minimum values observed across production-stage × analysis-unit comparisons (P1–P5 and pooled data). Route rankings included both self-loops and non-looping routes and were compared with the ≥10-s dataset without same-area merging. Non-looping transition counts were unchanged under every merge threshold.

Same-area merge rule	Same-area links merged, n (%)	Minimum Spearman route-rank correlation	Minimum top-20 route overlap
**No merging**	0 (0.00%)	1.000000	100%
**Gap ≤0 s**	110,342 (0.48%)	0.999954	100%
**Gap ≤5 s**	1,519,638 (6.66%)	0.999542	100%
**Gap ≤10 s**	**2,714,806 (11.90%)**	**0.998778**	**100%**
**Gap ≤15 s**	3,978,324 (17.44%)	0.998136	95%
**Gap ≤20 s**	5,083,504 (22.28%)	0.997632	95%
**Gap ≤25 s**	6,089,256 (26.69%)	0.996575	95%
**Gap ≤30 s**	6,986,289 (30.62%)	0.995869	95%
**Gap ≤45 s**	9,193,353 (40.29%)	0.994253	95%
**Gap ≤60 s**	10,889,996 (47.73%)	0.993809	95%

S2.5. Final Cleaning Rules

The final cleaning pipeline retained source bouts with durations ≥10 s and merged consecutive bouts assigned to the same functional area when their effective exit-to-next-entry gap was ≤10 s. These rules reduced 30,303,891 duration-qualified source bouts to 27,589,085 cleaned bouts before four-stage cohort restriction.

An A→B→A suppression rule was evaluated but not applied: only two of 1,345,814 such sequences after the 10-s duration filter met the additional <5-s entry-interval condition. The broader allocation-valid audit reported 15 different-area overlaps. The final 2,162-hen cohort contained 13 negative-gap non-looping pairs (five late-laying and eight end-laying); these distinct populations should not be conflated. Different-area overlaps were retained in entry-time order and excluded only in the nonnegative-gap sensitivities.

No maximum source-bout duration threshold was applied. Bouts exceeding 12 h were retained but flagged during quality control rather than removed using an arbitrary upper limit. Cleaning and transition construction were performed within each hen and calendar date, and sequences were restarted at the beginning of every retained date. Consequently, no transition was constructed across midnight, an excluded date, or a missing

source-file date. This defined the primary daily sequences; additional timing checks in Section S8.4 quantify eligible boundary-link additions without claiming reconstruction of continuous overnight use.

S3. Cohort Construction, Detection Coverage and Denominator Sensitivity

S3.1. Construction of the Final Analysis Cohort

The official flock roster contained 3,125 RFID-tagged hens, initially allocated equally among the five housing sections (625 hens per section). Post-rearrangement assignments were documented for 3,118 hens; seven hens without a documented new assignment were not eligible for analyses after rearrangement. Housing-section assignments were determined from the official flock records rather than inferred from RFID detections.

The hens were rearranged on the night of 19 December 2016. Records from this date were excluded. For retained dates up to 18 December, hens were assigned according to their original housing section (Old Pen). From 20 December onwards, they were assigned according to their post-rearrangement section (New Pen). The post-rearrangement allocation was P1 = stayers, P2 = roamers, P3 = rangers maintained under standard conditions, P4 = rangers provided with an outdoor range feeder, and P5 = rangers receiving the modified diet.

The final analysis cohort was defined as hens with at least one valid cleaned RFID bout on the retained recording dates in each of the four production stages. This was a stage-presence criterion; hens were not required to be detected on every retained date or on a predetermined percentage of dates. The resulting cohort contained 2,162 hens.

During pre-laying, the cohort comprised 447, 420, 393, 438 and 464 hens assigned to P1–P5, respectively, based on their original sections. Following rearrangement, the same cohort comprised 382, 453, 440, 445 and 442 hens in P1–P5, respectively, based on their new assignments (Table S4, panel A). Of the 2,162 hens, 444 (20.5%) received the same numbered section assignment before and after rearrangement, whereas 1,718 (79.5%) changed numbered sections.

Consequently, the pooled stage-level analyses follow the same individual cohort throughout the four stages, but a numbered section during pre-laying does not contain the same group of hens as that numbered section after rearrangement. Comparisons among peak-, late- and end-laying follow the established post-rearrangement groups, whereas comparisons between pre-laying and later stages should be interpreted as section-level rather than within-group longitudinal comparisons.

Cohort size was relatively insensitive to the recording-volume threshold used to select dates. In the preliminary raw-data audit, thresholds of 75%, 80% and 85% of the usual stage–section recording volume retained 204, 192 and 172 dates and identified 2,164, 2,164 and 2,163 candidate hens, respectively. After applying all final validity and cleaning rules to the selected 204 dates, 2,162 hens remained in the final analysis cohort.

Administrative status fields were also audited. Twenty cohort hens carried a DEAD (n = 8), LOST (n = 11) or BROKEN (n = 1) flag. A status date was available for 17 of these hens, and all 17 continued to produce valid RFID detections after the recorded date. These status fields were therefore not used to censor hen-day exposure or determine cohort membership.

S3.2. Detection Coverage

Two hen-day quantities were calculated. An **eligible hen-day** represented one cohort hen on one retained recording date, regardless of whether that hen produced a valid cleaned bout. A **detected hen-day** represented an eligible hen-day on which the hen produced at least one valid cleaned RFID bout. Detection coverage was calculated as:

$$\text{Detection coverage} = \frac{\text{detected hen-days}}{\text{eligible hen-days}}.$$

Pooled detection coverage declined across the production cycle, from 99.21% during pre-laying to 97.56% during peak-laying, 92.45% during late-laying and 84.19% during end-laying (Table S4, panel B). The percentage of hens detected on every retained date similarly declined from 97.18% during pre-laying to 54.30% during end-laying. Conversely, the number of hens detected on fewer than 50% of retained dates increased from 12 during pre-laying to 286 during end-laying.

Missing detected hen-days were concentrated among a minority of hens during the later stages. The lowest-coverage 10% of hens accounted for 69.2% of missing hen-days during late-laying and 51.1% during end-laying. Pairwise Pearson correlations between individual hens' coverage across stages were generally weak ($r = -0.027$ to 0.259), indicating that the hens with relatively low coverage were not consistently the same individuals in every stage.

S3.3. Denominator Sensitivity and Final Denominator Selection

Transition rates were calculated using both eligible and detected hen-days to evaluate the effect of the denominator. Both calculations used the same transition counts:

$$\text{Eligible-hen-day rate} = \frac{\text{number of transitions}}{\text{eligible hen-days}},$$

$$\text{Detected-hen-day rate} = \frac{\text{number of transitions}}{\text{detected hen-days}}.$$

The two rates were similar during pre- and peak-laying because detection coverage was high. Their difference increased during the later stages as the number of undetected hen-days increased (Table S4, panel C). Using detected rather than eligible hen-days increased both loop-inclusive and non-looping rates by 0.8% during pre-laying, 2.5% during peak-laying, 8.2% during late-laying and 18.8% during end-laying. Thus, rates based on eligible hen-days would increasingly treat days without usable RFID sequences as days with no transitions.

Excluding hens with incomplete detection coverage was considered as an alternative approach. Requiring each hen to be detected on at least 75% of the retained dates in every stage reduced the cohort from 2,162 to 1,480 hens, retaining only 68.5% of the final cohort. The percentage retained differed among the post-rearrangement sections, ranging from 59.7% in P5 to 79.5% in P2 (Table S4, panel D). This restriction would therefore have altered both cohort size and section composition.

Low-coverage hens could nevertheless produce informative transition sequences on the dates when they were detected. During end-laying, the 141 hens with coverage below 25% produced 14.52 non-looping transitions per detected hen-day, compared with 14.82 among the 1,174 hens detected on every retained date. Low detection coverage therefore did not necessarily indicate an absence of measurable movement on observed days.

Detected hen-days were consequently selected as the primary denominator. This denominator standardises transition counts by the number of hen-days that contributed at least one usable cleaned RFID bout, while retaining hens with intermittent but valid observations. It does not estimate movement on undetected days; rather, it describes the transition rate conditional on a hen contributing a valid sequence on that date. Unless otherwise stated, all transition rates reported in the main text and Supplementary Materials are expressed as transitions per detected hen-day. The same four-stage subset was also used to recompute rates (Table S9); no detection-probability correction was applied.

Table S4. Cohort composition, detection coverage and denominator sensitivity. Panel A shows the distribution of the final analysis cohort according to original and post-rearrangement housing-section assignments. Panel B summarises stage-level detection coverage. Panel C compares transition rates calculated using eligible and detected hen-days. Panel D shows the effect of requiring detection on at least 75% of retained dates in every stage.

Panel A. Final analysis cohort by housing-section assignment

Assignment basis	P1	P2	P3	P4	P5	Total
**Pre-laying: original section**	447	420	393	438	464	2,162
**Peak- to end-laying: post-rearrangement section**	382	453	440	445	442	2,162
**Same numbered section before and after rearrangement**	81	84	91	91	97	444

Panel B. Detection coverage by production stage

Stage	Retained dates	Eligible hen-days (retained date × total hens in each stage)	Detected hen-days	Detection coverage	Detected on every date, *n* (%)	Coverage <50%, *n*
**Pre-laying**	14	30,268	30,028	99.21%	2,101 (97.18%)	12
**Peak-laying**	27	58,374	56,951	97.56%	2,000 (92.51%)	32
**Late-laying**	83	179,446	165,900	92.45%	1,490 (68.92%)	65
**End-laying**	80	172,960	145,609	84.19%	1,174 (54.30%)	286

Panel C. Transition rates under alternative hen-day denominators

Stage	Loop-inclusive rate: eligible	Loop-inclusive rate: detected	Non-looping rate: eligible	Non-looping rate: detected	Increase using detected denominator
**Pre-laying**	54.46	54.90	9.57	9.65	0.8%
**Peak-laying**	56.85	58.27	13.21	13.54	2.5%
**Late-laying**	58.85	63.65	16.43	17.77	8.2%
**End-laying**	47.58	56.52	12.71	15.10	18.8%

Rates are expressed as transitions per eligible or detected hen-day, as indicated.

Panel D. Sensitivity to a ≥75% within-stage detection-coverage requirement

Cohort rule	P1	P2	P3	P4	P5	Total
**Final cohort: no additional date-coverage threshold**	382	453	440	445	442	2,162
**Coverage ≥75% in every stage**	275	360	281	300	264	1,480
**Percentage retained**	72.0%	79.5%	63.9%	67.4%	59.7%	68.5%

Panel D uses post-rearrangement section assignments to show how the additional coverage requirement would alter the composition of the established experimental groups.

S4. Self-Loop Gaps Between Consecutive Same-Area Detections

Self-loop gaps were summarised for the four-stage cohort of 2,162 hens, each of which contributed at least one valid cleaned RFID bout on the retained recording dates in every production stage. A self-loop was defined as two consecutive cleaned bouts from the same hen, within the same calendar day, that were assigned to the same functional area (A→A). For self-loop i, the gap was calculated as:

$$\text{Self-loop gap}_i = \text{Entry}_{i+1} - \text{Exit}_i$$

The self-loop gap therefore represents the interval from the end of one valid same-area detection bout to the beginning of the next. It does not represent the duration of either bout or demonstrate continuous occupancy of the area during the intervening period.

The hen's location during a self-loop gap was not observed. Consequently, a self-loop gap should not be interpreted as a continuous stay within the area or as confirmed movement away from and back to that area. The hen may have remained nearby without being detected, used an unmonitored location, or returned to the same monitored area without being detected elsewhere. Self-loop gaps were therefore interpreted as intervals between consecutive same-area detections rather than direct measures of area occupancy.

The final cleaning procedure retained antenna-level bouts lasting at least 10 s and merged consecutive bouts assigned to the same functional area when the effective exit-to-next-entry gap was ≤10 s. As expected, all remaining self-loop gaps were longer than 10 s, with a minimum of 11 s (0.18 min) in every production stage. Self-loops were constructed only within hen × calendar day, so no interval crossed midnight or an excluded recording date. No upper-duration cap was applied.

A total of 17,549,442 self-loops contributed to the summaries (Table S5A). The raw numbers differed considerably among stages because the stages contained different numbers of retained recording dates and detected hen-days. Self-loop activity was therefore compared using rates per detected hen-day rather than raw event counts. Rates were similar during pre-, peak- and late-laying, at 45.25, 44.72 and 45.88 loops per detected hen-day, respectively, before decreasing to 41.43 during end-laying. Thus, the increase in total loop-inclusive transition activity from pre- to late-laying was largely associated with increasing non-looping movement, while the frequency of self-loops remained broadly stable.

Self-loop gaps were strongly right-skewed in every stage. Median gaps ranged from 1.30 to 1.65 min, whereas means ranged from 5.53 to 6.19 min. Median gaps were descriptively longest during peak-laying and shorter during late- and end-laying. Nevertheless, the overall differences were modest: 75% of gaps were shorter than approximately 3.5–4.7 min across stages, and the 95th percentiles were consistently approximately 27–29 min.

Long gaps formed only a very small part of the distributions. Of the 17,549,442 self-loops, 3,531 (0.020%) had gaps longer than 4 h, and only eight had gaps longer than 12 h. These extreme values had little influence on the medians and interquartile ranges but contributed to the higher means and maximum values. Because these gaps represent unobserved intervals rather than continuous stays, they were retained without truncation.

Functional-area distribution

Self-loops occurred overwhelmingly at feeder locations (Table S5B). Self-loops at the lower and upper feeders in Rows 1 and 2 together accounted for 99.53% of all self-loops during pre-laying, declining descriptively to 94.99% during end-laying. Nest-box self-loops remained uncommon throughout the study, accounting for only 0.32–0.54% of stage-specific self-loops.

In contrast, the combined contribution of the outdoor-interface areas—Pop-hole, Range and range feeder—increased from 0.09% during pre-laying to 1.13% during peak-laying, 3.78% during late-laying and 4.47% during end-laying. Most of this later-stage increase occurred at the Pop-hole and range feeder nodes. Range feeder was

present only in P4, so its contribution represents a housing-section-specific feature rather than a resource available across all five sections.

Nest-box self-loop gaps had medians of approximately 1.5–2.8 min, depending on row and stage. These values do not represent nest occupancy or time required for oviposition; they describe only the interval between two consecutive nest-box detections. Similarly, the longer gaps observed for some Pop-hole and Range self-loops should not be interpreted as uninterrupted time spent outdoors.

Housing-section pattern

Section-specific summaries showed a consistent descriptive difference for P5 (Table S5C). Across stages, P5 had fewer self-loops per detected hen-day and longer median gaps than P1–P4. P5 rates ranged from 23.20 to 27.89 loops per detected hen-day, whereas the corresponding ranges across P1–P4 were 35.46–65.13. Median gaps in P5 ranged from 1.93 to 3.05 min, compared with 1.15–1.65 min across P1–P4.

This difference was present both before and after the flock rearrangement, when the hens occupying the sections changed. It should therefore not be attributed directly to the post-rearrangement behavioural allocation or modified diet in P5. The persistent pattern may instead reflect a physical-section, antenna-availability or detection-system feature, although the present descriptive analysis cannot distinguish among these possibilities.

Table S5. Self-loop gaps between consecutive same-area detections after final RFID cleaning. A self-loop gap was calculated as the entry time of the subsequent bout minus the exit time of the preceding bout for two adjacent cleaned bouts assigned to the same functional area. Gaps are reported in minutes. Pooled summaries concatenate individual self-loop events across P1–P5 and are therefore weighted by the number of self-loops rather than giving equal weight to each housing section. Rates are expressed per detected hen-day. Self-loop gaps are unobserved intervals and should not be interpreted as continuous area stays.

A. Pooled self-loop gap summaries by production stage

Stage	*n* self-loops	Self-loops per detected hen-day	Minimum	Q1	Median	Q3	Mean (SD)	P95	P99	Maximum
Pre-laying	1,358,787	45.25	0.18	0.62	1.53	4.50	6.18 (15.91)	27.95	79.42	752.40
Peak-laying	2,547,117	44.72	0.18	0.67	1.65	4.72	6.19 (14.93)	28.55	74.56	441.08
Late-laying	7,611,627	45.88	0.18	0.58	1.33	3.58	5.53 (14.36)	27.23	70.22	917.23
End-laying	6,031,911	41.43	0.18	0.57	1.30	3.48	5.83 (15.78)	29.37	78.83	1,408.43

B. Percentage distribution of self-loops among functional-area categories

Stage	Feeder	Nest box	Pop-hole	Range	Range feeder
**Pre-laying**	99.53%	0.38%	0.03%	<0.01%	0.06%
**Peak-laying**	98.55%	0.32%	0.61%	<0.01%	0.52%
**Late-laying**	95.83%	0.40%	2.59%	0.01%	1.18%
**End-laying**	94.99%	0.54%	2.92%	0.02%	1.54%

Feeder includes lower- and upper-feeder areas in both aviary rows (R1:LF, R1:UF, R2:LF and R2:UF). Nest box includes R1:NB and R2:NB. Pop-hole includes the readers mapped to the common Pop-hole functional node but does not include Range or range feeder. Range feeder was present only in P4. Percentages may not total exactly 100% because of rounding.

C. Descriptive comparison of P5 with P1–P4

Stage	Median gap range in P1–P4 (min)	P5 median gap (min)	Loop-rate range in P1–P4	P5 loop rate
**Pre-laying**	1.37–1.60	3.05	46.81–53.61	23.20
**Peak-laying**	1.47–1.65	2.93	42.43–60.24	26.41
**Late-laying**	1.20–1.32	2.32	40.41–65.13	27.59
**End-laying**	1.15–1.35	1.93	35.46–56.87	27.89

Loop rates in Panel C are expressed as self-loops per detected hen-day. Pre-laying housing-section summaries use the original section allocation, whereas peak-, late- and end-laying summaries use the post-rearrangement allocation. The P5 comparison is descriptive and does not establish a treatment or behavioural-group effect.

S5. Housing-Section-Level Top 20 Transitions

Table S6 presents the 20 highest-ranked loop-inclusive routes within each housing section and production stage for the final four-stage cohort. Routes were ranked separately within each section–stage combination. Route-specific rates were calculated by dividing transition counts by the total number of detected hen-days within the corresponding housing section and stage.

P1–P5 identify physical housing sections. Pre-laying records reflect the original allocation, whereas peak-, late- and end-laying records reflect the post-rearrangement allocation. The same housing-section label therefore does not imply unchanged hen membership across rearrangement.

Table S6 (A–E). Twenty highest-rate loop-inclusive transitions within each housing section across four production stages. Rates are expressed as transitions per detected hen-day, and self-loops are shown in bold. Location labels follow the Row notation: R1 and R2 identify aviary rows; LF, UF and NB denote lower feeders, upper feeders and nest boxes, respectively. RF denotes the outdoor range feeder in P4. The absence of a route from these rankings does not indicate that its transition count was zero. Complete transition matrices are presented in the housing-section-level heatmaps in Supplementary Section S6.

A) Top-20 loop-inclusive transitions - Housing section P1

Rank	Pre-laying			Peak-laying			Late-laying			End-laying		
Rank	From	To	Rate	From	To	Rate	From	To	Rate	From	To	Rate
1	**R2:LF**	**R2:LF**	**14.29**	**R2:UF**	**R2:UF**	**17.20**	**R2:UF**	**R2:UF**	**18.74**	**R1:UF**	**R1:UF**	**17.29**
2	**R2:UF**	**R2:UF**	**12.68**	**R1:UF**	**R1:UF**	**16.40**	**R1:UF**	**R1:UF**	**18.40**	**R1:LF**	**R1:LF**	**13.85**
3	**R1:LF**	**R1:LF**	**12.36**	**R2:LF**	**R2:LF**	**14.31**	**R1:LF**	**R1:LF**	**13.33**	**R2:UF**	**R2:UF**	**13.06**
4	**R1:UF**	**R1:UF**	**12.05**	**R1:LF**	**R1:LF**	**11.96**	**R2:LF**	**R2:LF**	**13.08**	**R2:LF**	**R2:LF**	**11.22**
5	Pop-hole	R2:LF	1.23	R1:LF	R2:LF	1.13	Pop-hole	R2:LF	2.01	Pop-hole	R2:LF	1.63
6	R2:LF	Pop-hole	1.20	R2:LF	R1:LF	1.05	R2:LF	Pop-hole	1.93	R2:LF	Pop-hole	1.42
7	R2:LF	R1:LF	1.11	R2:LF	Pop-hole	0.99	Pop-hole	R1:LF	1.62	R1:LF	Pop-hole	1.26
8	R1:LF	R2:LF	1.10	Pop-hole	R2:LF	0.99	R1:LF	Pop-hole	1.55	**Pop-hole**	**Pop-hole**	**1.21**
9	R1:LF	Pop-hole	1.01	Pop-hole	R1:LF	0.82	**Pop-hole**	**Pop-hole**	**1.42**	Pop-hole	R1:LF	1.18
10	Pop-hole	R1:LF	0.98	R1:NB	R1:UF	0.75	R1:LF	R2:LF	1.10	R2:LF	R1:LF	1.00
11	R1:NB	R1:UF	0.29	R1:LF	Pop-hole	0.74	R2:LF	R1:LF	1.08	R1:UF	R1:NB	0.90
12	R2:NB	R2:UF	0.29	R1:UF	R1:NB	0.68	R1:NB	R1:UF	0.91	R1:NB	R1:UF	0.90
13	R1:LF	R1:NB	0.28	R2:NB	R2:UF	0.68	R2:UF	R2:NB	0.90	R1:LF	R2:LF	0.87
14	R2:LF	R2:NB	0.26	R2:UF	R2:NB	0.63	R2:NB	R2:UF	0.89	R2:UF	R2:NB	0.77
15	R2:UF	R2:NB	0.23	R1:LF	R1:NB	0.30	R1:UF	R1:NB	0.86	R2:NB	R2:UF	0.73
16	R1:NB	R1:LF	0.21	R2:LF	R2:NB	0.28	R1:LF	R1:NB	0.32	R1:LF	R1:NB	0.32
17	R1:UF	R1:NB	0.21	R1:NB	R1:LF	0.26	R2:LF	R2:NB	0.28	R1:NB	R1:LF	0.26
18	R2:NB	R2:LF	0.21	R2:NB	R2:LF	0.23	R1:NB	R1:LF	0.26	R2:LF	R2:NB	0.25
19	R2:LF	R2:UF	0.12	**Pop-hole**	**Pop-hole**	**0.22**	R2:NB	R2:LF	0.24	R2:NB	R2:LF	0.21
20	**R1:NB**	**R1:NB**	**0.11**	R2:LF	R2:UF	0.20	R2:LF	R2:UF	0.21	**R1:NB**	**R1:NB**	**0.21**

B) Top-20 loop-inclusive transitions - Housing section P2

Rank	Pre-laying			Peak-laying			Late-laying			End-laying		
Rank	From	To	Rate	From	To	Rate	From	To	Rate	From	To	Rate
1	**R2:LF**	**R2:LF**	**16.94**	**R2:LF**	**R2:LF**	**14.28**	**R2:LF**	**R2:LF**	**13.51**	**R1:LF**	**R1:LF**	**12.73**
2	**R1:LF**	**R1:LF**	**14.24**	**R1:LF**	**R1:LF**	**12.34**	**R2:UF**	**R2:UF**	**12.85**	**R2:LF**	**R2:LF**	**12.22**
3	**R2:UF**	**R2:UF**	**11.16**	**R2:UF**	**R2:UF**	**11.96**	**R1:LF**	**R1:LF**	**12.25**	**R2:UF**	**R2:UF**	**9.25**
4	**R1:UF**	**R1:UF**	**10.94**	**R1:UF**	**R1:UF**	**9.25**	**R1:UF**	**R1:UF**	**8.83**	**R1:UF**	**R1:UF**	**7.60**
5	R2:LF	R1:LF	1.21	R1:LF	R2:LF	1.64	Pop-hole	R2:LF	2.38	Pop-hole	R2:LF	1.82
6	R1:LF	R2:LF	1.20	R2:LF	R1:LF	1.57	R2:LF	Pop-hole	2.24	R2:LF	Pop-hole	1.58
7	Pop-hole	R2:LF	0.88	Pop-hole	R2:LF	1.30	Pop-hole	R1:LF	1.80	R1:LF	Pop-hole	1.41
8	R2:LF	Pop-hole	0.85	R2:LF	Pop-hole	1.27	R1:LF	Pop-hole	1.77	R2:LF	R1:LF	1.36
9	R1:LF	Pop-hole	0.62	Pop-hole	R1:LF	1.01	**Pop-hole**	**Pop-hole**	**1.56**	Pop-hole	R1:LF	1.32
10	Pop-hole	R1:LF	0.62	R1:LF	Pop-hole	0.94	R2:LF	R1:LF	1.51	**Pop-hole**	**Pop-hole**	**1.24**
11	R2:LF	R2:NB	0.41	R2:NB	R2:UF	0.63	R1:LF	R2:LF	1.50	R1:LF	R2:LF	1.20
12	R2:NB	R2:LF	0.33	R2:UF	R2:NB	0.61	R2:UF	R2:NB	0.70	R2:UF	R2:NB	0.65
13	R2:NB	R2:UF	0.33	R2:LF	R2:NB	0.47	R2:NB	R2:UF	0.69	R2:NB	R2:UF	0.62
14	**R2:NB**	**R2:NB**	**0.30**	R1:NB	R1:UF	0.47	R2:LF	R2:NB	0.52	R1:NB	R1:UF	0.45
15	R2:UF	R2:NB	0.27	R1:UF	R1:NB	0.38	R1:NB	R1:UF	0.48	R2:LF	R2:NB	0.45
16	R1:LF	R1:NB	0.21	R2:NB	R2:LF	0.38	R2:NB	R2:LF	0.45	R1:UF	R1:NB	0.41
17	R1:NB	R1:UF	0.20	**Pop-hole**	**Pop-hole**	**0.30**	R1:UF	R1:NB	0.42	R2:NB	R2:LF	0.38
18	R1:NB	R1:LF	0.15	R1:LF	R1:NB	0.26	**R2:NB**	**R2:NB**	**0.32**	**R2:NB**	**R2:NB**	**0.37**
19	R1:UF	R1:NB	0.13	R2:LF	R2:UF	0.26	R2:LF	R2:UF	0.28	R1:LF	R1:NB	0.28
20	R1:LF	R1:UF	0.12	**R2:NB**	**R2:NB**	**0.24**	R1:LF	R1:NB	0.27	R1:NB	R1:LF	0.19

C) Top-20 loop-inclusive transitions - Housing section P3

**Rank**	**Pre-laying**			**Peak-laying**			**Late-laying**			**End-laying**		
**Rank**	**From**	**To**	**Rate**	**From**	**To**	**Rate**	**From**	**To**	**Rate**	**From**	**To**	**Rate**
1	**R2:LF**	**R2:LF**	**14.02**	**R2:LF**	**R2:LF**	**17.01**	**R2:LF**	**R2:LF**	**14.65**	**R2:LF**	**R2:LF**	**12.18**
2	**R1:LF**	**R1:LF**	**12.38**	**R1:LF**	**R1:LF**	**11.73**	**R1:LF**	**R1:LF**	**12.56**	**R1:LF**	**R1:LF**	**12.08**
3	**R2:UF**	**R2:UF**	**11.12**	**R2:UF**	**R2:UF**	**8.19**	**R2:UF**	**R2:UF**	**7.85**	**R2:UF**	**R2:UF**	**5.91**
4	**R1:UF**	**R1:UF**	**9.20**	**R1:UF**	**R1:UF**	**5.33**	**R1:UF**	**R1:UF**	**4.97**	**R1:UF**	**R1:UF**	**4.31**
5	R2:LF	R1:LF	1.25	R1:LF	R2:LF	1.99	R2:LF	R1:LF	1.99	Pop-hole	R2:LF	1.91
6	R1:LF	R2:LF	1.23	R2:LF	R1:LF	1.92	R1:LF	R2:LF	1.97	R2:LF	Pop-hole	1.68
7	Pop-hole	R2:LF	0.89	Pop-hole	R2:LF	1.43	Pop-hole	R2:LF	1.85	R2:LF	R1:LF	1.62
8	R2:LF	Pop-hole	0.86	R2:LF	Pop-hole	1.42	R2:LF	Pop-hole	1.76	R1:LF	R2:LF	1.44
9	R1:LF	Pop-hole	0.67	Pop-hole	R1:LF	0.97	Pop-hole	R1:LF	1.28	R1:LF	Pop-hole	1.40
10	Pop-hole	R1:LF	0.66	R1:LF	Pop-hole	0.91	R1:LF	Pop-hole	1.28	Pop-hole	R1:LF	1.28
11	R2:LF	R2:NB	0.30	R2:NB	R2:UF	0.53	R2:UF	R2:NB	0.66	**Pop-hole**	**Pop-hole**	**0.81**
12	R2:NB	R2:UF	0.28	R2:UF	R2:NB	0.48	R2:NB	R2:UF	0.66	R2:UF	R2:NB	0.60
13	R2:NB	R2:LF	0.23	R2:LF	R2:NB	0.43	R2:LF	R2:NB	0.54	R2:NB	R2:UF	0.59
14	R1:NB	R1:UF	0.22	R1:NB	R1:UF	0.35	R2:NB	R2:LF	0.43	R2:LF	R2:NB	0.47
15	R2:UF	R2:NB	0.22	R2:NB	R2:LF	0.30	R1:NB	R1:UF	0.34	R2:NB	R2:LF	0.36
16	R1:LF	R1:NB	0.22	R2:LF	R2:UF	0.29	R1:UF	R1:NB	0.28	R1:NB	R1:UF	0.36
17	R1:UF	R1:NB	0.16	R1:UF	R1:NB	0.25	**Pop-hole**	**Pop-hole**	**0.26**	R1:LF	R1:NB	0.29
18	R1:NB	R1:LF	0.15	R1:LF	R1:UF	0.23	R1:LF	R1:NB	0.26	R1:UF	R1:NB	0.28
19	R1:LF	R1:UF	0.12	R1:LF	R1:NB	0.22	R2:LF	R2:UF	0.22	R2:NB	R1:LF	0.17
20	R2:LF	R2:UF	0.12	R2:UF	R2:LF	0.21	R1:LF	R1:UF	0.19	R1:NB	R1:LF	0.17

D) Top-20 loop-inclusive transitions - Housing section P4

Rank	Pre-laying			Peak-laying			Late-laying			End-laying		
**Rank**	**From**	**To**	**Rate**	**From**	**To**	**Rate**	**From**	**To**	**Rate**	**From**	**To**	**Rate**
1	**R1:LF**	**R1:LF**	**14.45**	**R2:LF**	**R2:LF**	**15.17**	**R1:LF**	**R1:LF**	**14.66**	**R1:LF**	**R1:LF**	**14.04**
2	**R2:UF**	**R2:UF**	**13.35**	**R1:LF**	**R1:LF**	**14.54**	**R2:LF**	**R2:LF**	**12.99**	**R2:LF**	**R2:LF**	**11.52**
3	**R2:LF**	**R2:LF**	**13.33**	**R2:UF**	**R2:UF**	**9.55**	**R2:UF**	**R2:UF**	**9.85**	**R2:UF**	**R2:UF**	**8.51**
4	**R1:UF**	**R1:UF**	**11.34**	**R1:UF**	**R1:UF**	**7.11**	**R1:UF**	**R1:UF**	**6.63**	**R1:UF**	**R1:UF**	**6.51**
5	R1:LF	R2:LF	1.22	R1:LF	R2:LF	1.92	**RF**	**RF**	**2.65**	**RF**	**RF**	**3.12**
6	R2:LF	R1:LF	1.21	R2:LF	R1:LF	1.82	Pop-hole	R2:LF	2.54	Pop-hole	R2:LF	2.27
7	R2:LF	Pop-hole	0.93	Pop-hole	R2:LF	1.56	R2:LF	Pop-hole	2.40	R2:LF	Pop-hole	1.95
8	Pop-hole	R2:LF	0.93	R2:LF	Pop-hole	1.54	Pop-hole	R1:LF	1.85	R1:LF	Pop-hole	1.73
9	Pop-hole	R1:LF	0.70	Pop-hole	R1:LF	1.14	R1:LF	Pop-hole	1.82	Pop-hole	R1:LF	1.62
10	R1:LF	Pop-hole	0.69	**RF**	**RF**	**1.14**	R2:LF	R1:LF	1.64	**Pop-hole**	**Pop-hole**	**1.58**
11	R1:LF	R1:NB	0.33	R1:LF	Pop-hole	1.06	R1:LF	R2:LF	1.61	R2:LF	R1:LF	1.33
12	R1:NB	R1:UF	0.33	R1:NB	R1:UF	0.45	**Pop-hole**	**Pop-hole**	**1.43**	R1:LF	R2:LF	1.12
13	R2:LF	R2:NB	0.29	R2:NB	R2:UF	0.40	R2:NB	R2:UF	0.53	Range	RF	0.52
14	R2:NB	R2:UF	0.27	R1:UF	R1:NB	0.38	R2:UF	R2:NB	0.49	RF	Range	0.50
15	R1:UF	R1:NB	0.27	R2:UF	R2:NB	0.37	R1:NB	R1:UF	0.45	R2:NB	R2:UF	0.49
16	R1:NB	R1:LF	0.26	R2:LF	R2:NB	0.34	R1:LF	R1:NB	0.44	R2:UF	R2:NB	0.47
17	R2:UF	R2:NB	0.21	**Pop-hole**	**Pop-hole**	**0.33**	R2:LF	R2:NB	0.40	R1:LF	R1:NB	0.43
18	R2:NB	R2:LF	0.20	R1:LF	R1:NB	0.32	R1:UF	R1:NB	0.39	R1:NB	R1:UF	0.43
19	**RF**	**RF**	**0.14**	R2:LF	R2:UF	0.24	Range	RF	0.38	RF	Pop-hole	0.39
20	R1:LF	R1:UF	0.14	R2:NB	R2:LF	0.24	RF	Pop-hole	0.37	R1:UF	R1:NB	0.38

E) Top-20 loop-inclusive transitions - Housing section P5

Rank	Pre-laying			Peak-laying			Late-laying			End-laying		
**Rank**	**From**	**To**	**Rate**	**From**	**To**	**Rate**	**From**	**To**	**Rate**	**From**	**To**	**Rate**
1	**R2:LF**	**R2:LF**	**8.28**	**R1:LF**	**R1:LF**	**9.84**	**R2:LF**	**R2:LF**	**10.46**	**R2:LF**	**R2:LF**	**11.30**
2	**R1:LF**	**R1:LF**	**8.01**	**R2:LF**	**R2:LF**	**9.74**	**R1:LF**	**R1:LF**	**8.67**	**R1:LF**	**R1:LF**	**8.94**
3	**R2:UF**	**R2:UF**	**3.40**	**R2:UF**	**R2:UF**	**3.31**	**R2:UF**	**R2:UF**	**3.74**	**R2:UF**	**R2:UF**	**3.66**
4	**R1:UF**	**R1:UF**	**3.37**	**R1:UF**	**R1:UF**	**3.00**	**R1:UF**	**R1:UF**	**3.30**	**R1:UF**	**R1:UF**	**2.67**
5	Pop-hole	R2:LF	1.25	R2:LF	R1:LF	1.51	Pop-hole	R2:LF	2.32	Pop-hole	R2:LF	2.25
6	R2:LF	Pop-hole	1.20	R1:LF	R2:LF	1.48	R2:LF	Pop-hole	2.25	R2:LF	Pop-hole	2.00
7	R2:LF	R1:LF	1.11	Pop-hole	R2:LF	1.36	Pop-hole	R1:LF	1.66	R1:LF	Pop-hole	1.33
8	R1:LF	R2:LF	1.10	R2:LF	Pop-hole	1.32	R1:LF	R2:LF	1.60	Pop-hole	R1:LF	1.29
9	R1:LF	Pop-hole	0.99	Pop-hole	R1:LF	1.11	R2:LF	R1:LF	1.51	R2:LF	R1:LF	1.23
10	Pop-hole	R1:LF	0.97	R1:LF	Pop-hole	1.05	R1:LF	Pop-hole	1.49	**Pop-hole**	**Pop-hole**	**1.20**
11	R1:LF	R1:NB	0.50	R1:LF	R1:NB	0.58	**Pop-hole**	**Pop-hole**	**1.28**	R1:LF	R2:LF	1.07
12	R1:NB	R1:LF	0.37	R1:NB	R1:LF	0.40	R1:NB	R1:UF	0.45	R2:LF	R2:UF	0.37
13	R2:LF	R2:NB	0.27	R1:NB	R1:UF	0.40	R1:LF	R1:NB	0.42	R1:NB	R1:UF	0.34
14	R1:NB	R1:UF	0.26	**Pop-hole**	**Pop-hole**	**0.38**	R2:LF	R2:UF	0.41	R2:NB	R2:UF	0.33
15	R1:UF	R1:NB	0.20	R1:UF	R1:NB	0.30	R1:UF	R1:NB	0.37	R1:LF	R1:NB	0.32
16	R2:LF	R2:UF	0.17	R2:LF	R2:UF	0.30	R2:NB	R2:UF	0.34	R2:LF	R2:NB	0.30
17	R2:NB	R2:UF	0.15	R2:NB	R2:UF	0.30	R1:LF	R1:UF	0.33	R2:UF	R2:NB	0.28
18	R2:NB	R2:LF	0.15	R1:LF	R1:UF	0.28	R2:UF	R2:NB	0.29	R1:UF	R1:NB	0.27
19	R1:NB	R2:LF	0.13	R2:LF	R2:NB	0.25	R2:UF	Pop-hole	0.28	R1:LF	R1:UF	0.22
20	R1:LF	R1:UF	0.13	R2:UF	R1:LF	0.22	R2:UF	R1:LF	0.28	R2:UF	R2:LF	0.21

S6. Housing Section Transition Heatmaps

Figures S1–S5 group the four stages for each physical section. Rows are origins, columns destinations, and diagonal cells are self-loops. Rates use the section-stage detected hen-day denominator. The colour scale is shared with the four pooled matrices in main Figure 3. Selected route rates are also reported in the top-transition tables in Section S5; the section-stage denominator is defined in Section S3.

Grey zero cells mean no recorded transition for a represented pair. NR with hatching marks the absence of a regular Range stream in P2, P3 and P5 during pre- and peak-laying; these cells are not comparable measured zeros of range use. Late-laying Range cells in those sections are hatched and labelled with their 27 April 2017 stream start. These flags describe the available recording streams, not the dates when physical range access began or independent proof of hardware installation. RF is shown only in P4, using its own denominator.

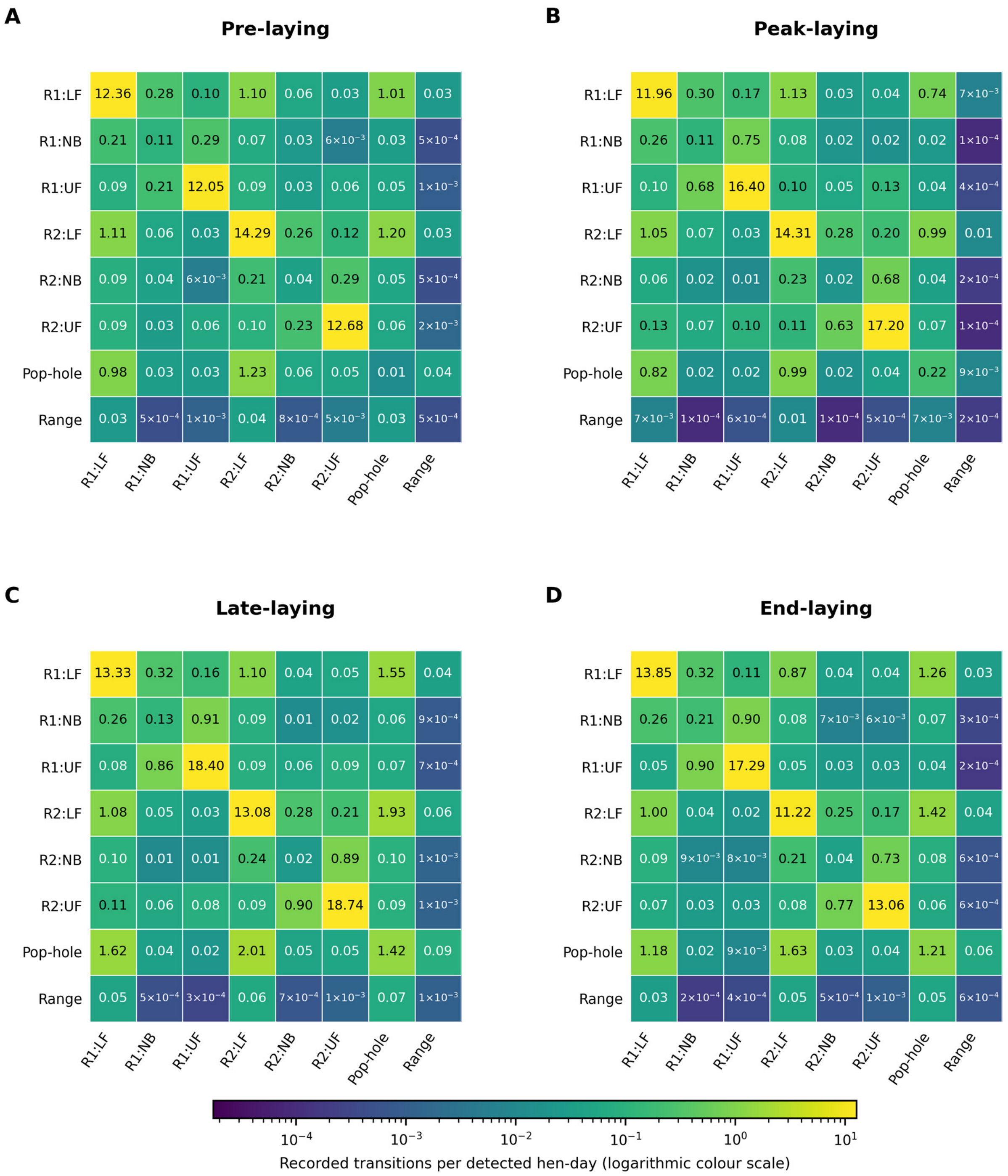

Figure S1. Recorded transitions in P1: (A) pre-, (B) peak-, (C) late- and (D) end-laying. Cells show transitions per detected hen-day on a shared logarithmic colour scale. Rows are origins and columns destinations; diagonals are self-loops. Grey zeros indicate no recorded transition for represented pairs. NR (no regular recording stream), hatching and dagger notes identify absent or partial regular Range streams. RF is structurally absent outside P4 and is omitted from those matrices. R1/R2, aviary rows; LF, lower feeder; NB, nest box; UF, upper feeder; RF, outdoor range feeder. Numbered sections changed composition after pre-laying.

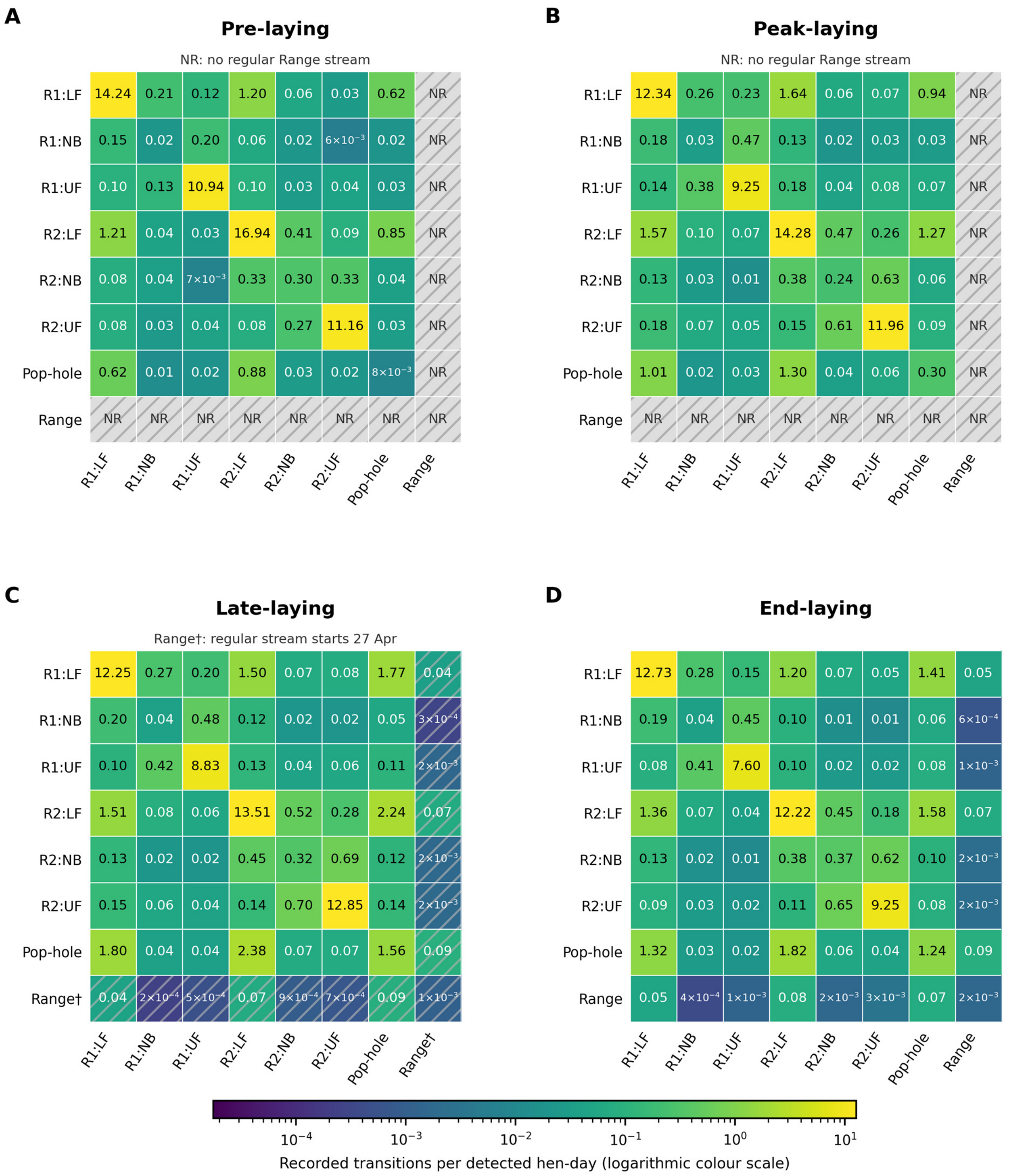

Figure S2. Recorded transitions in P2: (A) pre-, (B) peak-, (C) late- and (D) end-laying. Cells show transitions per detected hen-day on a shared logarithmic colour scale. Rows are origins and columns destinations; diagonals are self-loops. Grey zeros indicate no recorded transition for represented pairs. NR (no regular recording stream), hatching and dagger notes identify absent or partial regular Range streams. RF is structurally absent outside P4 and is omitted from those matrices. R1/R2, aviary rows; LF, lower feeder; NB, nest box; UF, upper feeder; RF, outdoor range feeder. Numbered sections changed composition after pre-laying.

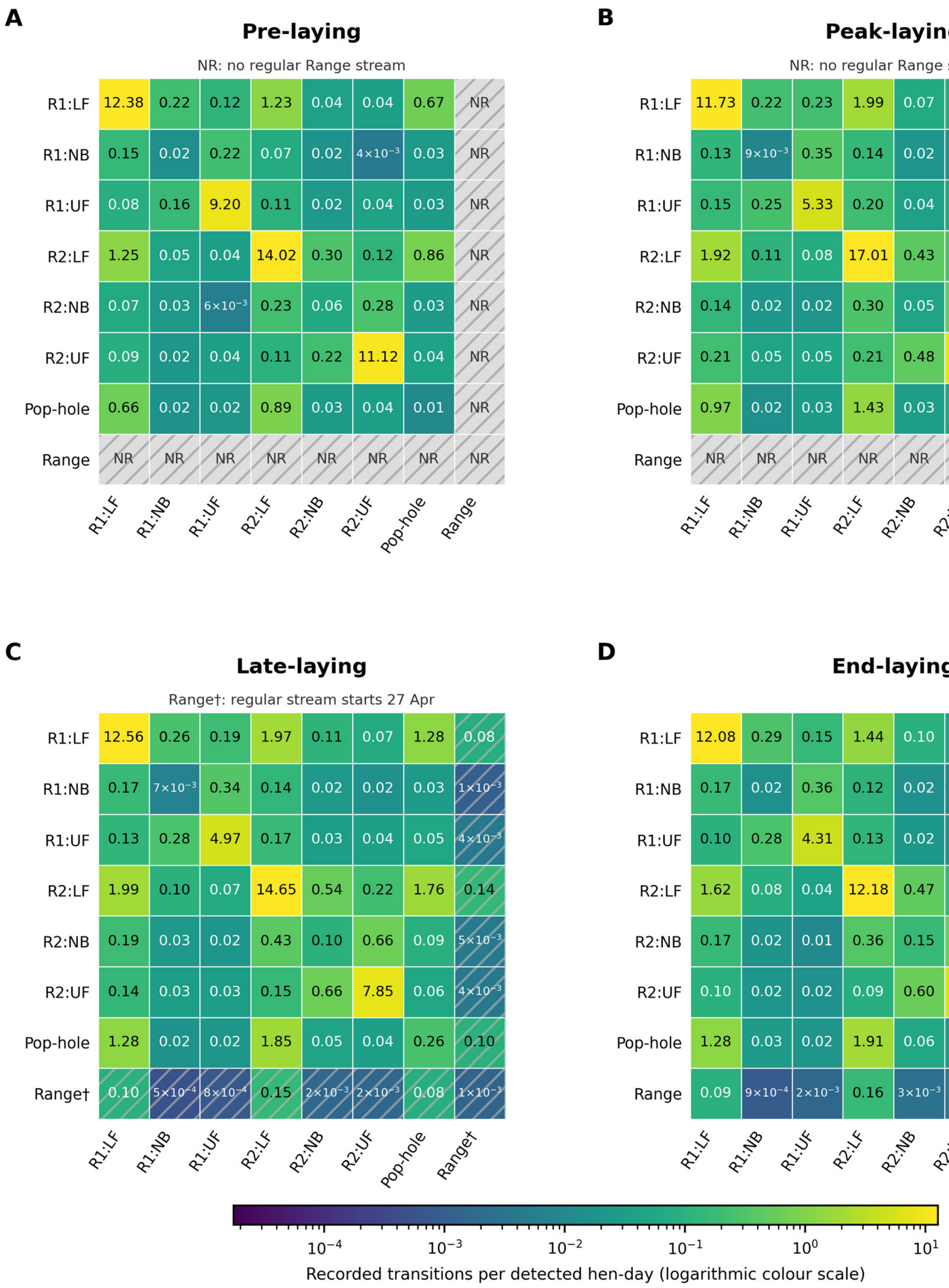

Figure S3. Recorded transitions in P3: (A) pre-, (B) peak-, (C) late- and (D) end-laying. Cells show transitions per detected hen-day on a shared logarithmic colour scale. Rows are origins and columns destinations; diagonals are self-loops. Grey zeros indicate no recorded transition for represented pairs. NR (no regular recording stream), hatching and dagger notes identify absent or partial regular Range streams. RF is structurally absent outside P4 and is omitted from those matrices. R1/R2, aviary rows; LF, lower feeder; NB, nest box; UF, upper feeder; RF, outdoor range feeder. Numbered sections changed composition after pre-laying.

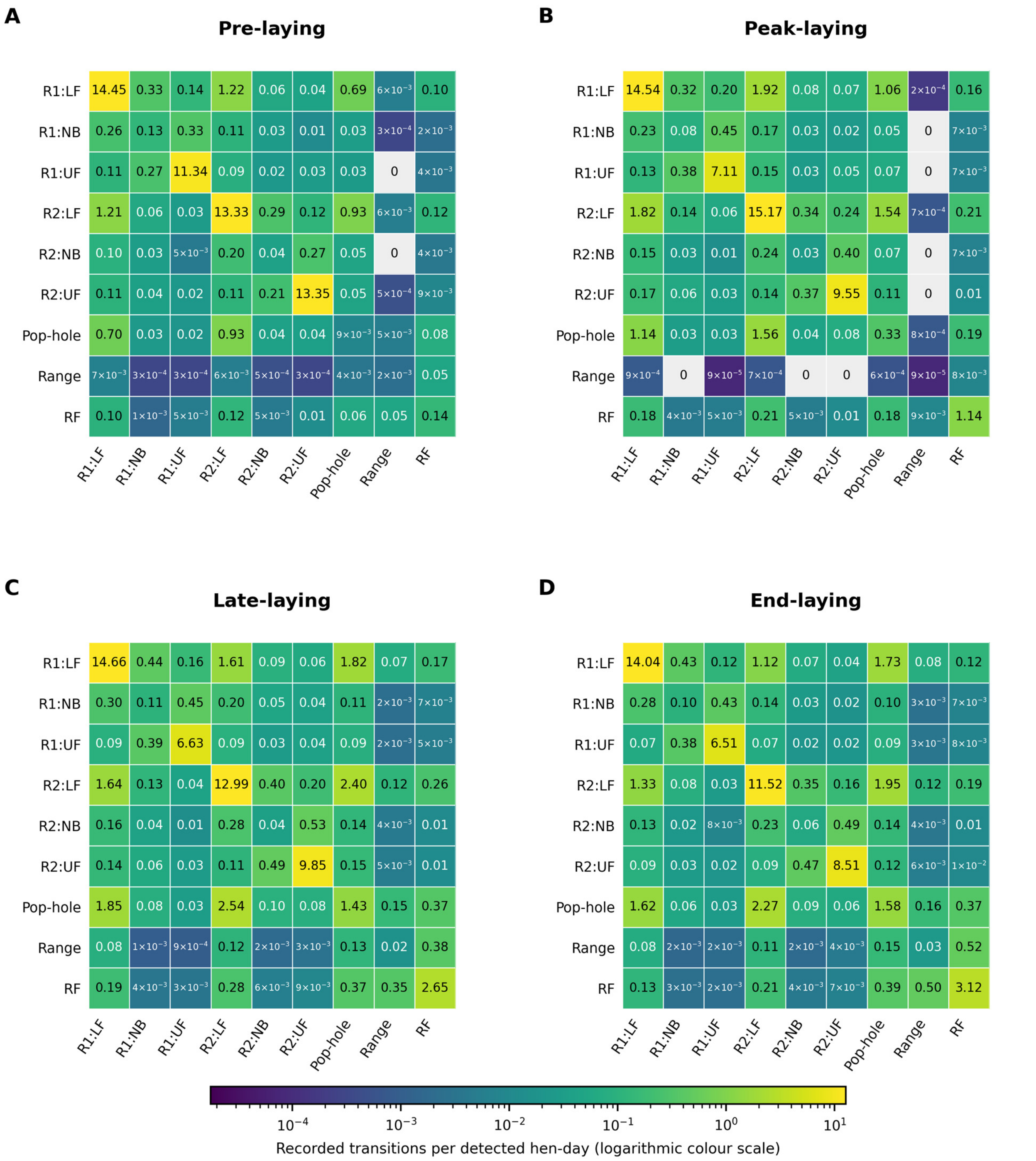

Figure S4. Recorded transitions in P4: (A) pre-, (B) peak-, (C) late- and (D) end-laying. Cells show transitions per detected hen-day on a shared logarithmic colour scale. Rows are origins and columns destinations; diagonals are self-loops. Grey zeros indicate no recorded transition for represented pairs. NR (no regular recording stream), hatching and dagger notes identify absent or partial regular Range streams. RF is structurally absent outside P4 and is omitted from those matrices. R1/R2, aviary rows; LF, lower feeder; NB, nest box; UF, upper feeder; RF, outdoor range feeder. Numbered sections changed composition after pre-laying.

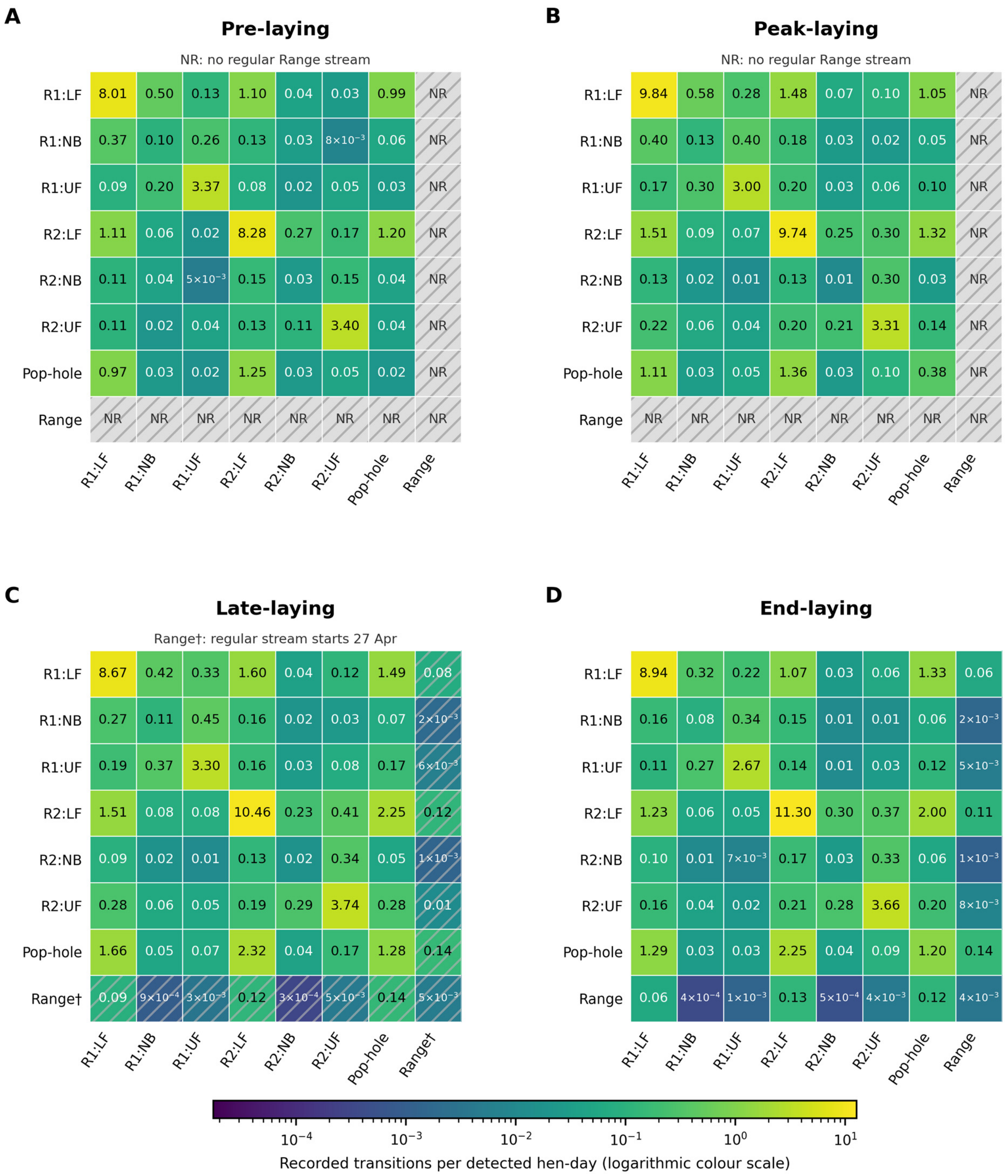

Figure S5. Recorded transitions in P5: (A) pre-, (B) peak-, (C) late- and (D) end-laying. Cells show transitions per detected hen-day on a shared logarithmic colour scale. Rows are origins and columns destinations; diagonals are self-loops. Grey zeros indicate no recorded transition for represented pairs. NR (no regular recording stream), hatching and dagger notes identify absent or partial regular Range streams. RF is structurally absent outside P4 and is omitted from those matrices. R1/R2, aviary rows; LF, lower feeder; NB, nest box; UF, upper feeder; RF, outdoor range feeder. Numbered sections changed composition after pre-laying.

S7. Interactive Transition Diagrams

The accompanying offline interactive file contains 48 selections: four stages × six analysis units (P1–P5 and pooled P1–P5) × two representations (loop-inclusive and non-looping). It opens without an external website or internet connection. Left and right columns repeat location labels as origins and destinations; same-area ribbons are self-loops. Ribbons describe adjacent recorded bouts and do not establish a continuous physical path.

Positive routes at or above the within-selection 80th percentile of counts are displayed, with ties included. Self-loops are removed before calculating the threshold in the non-looping representation. Pooled views omit RF-linked routes to avoid displaying a P4-only resource against pooled exposure; P4 views retain them using P4 detected hen-days. The filtering threshold is calculated within the selected displayed network. Ribbon widths are independently scaled, and numerical rates should be used for comparisons. Hover labels and the displayed table give counts, rates and detected hen-days.

Coverage notes are displayed with every selection. Omitted routes need not have zero counts, and no regular Range stream is not evidence of absent physical range use. No individual tag histories or raw reader records are included. Main Figure 3 provides complete pooled common-location matrices. Figure S6 supplies a fixed route comparison to avoid relying on independently scaled Sankey widths.

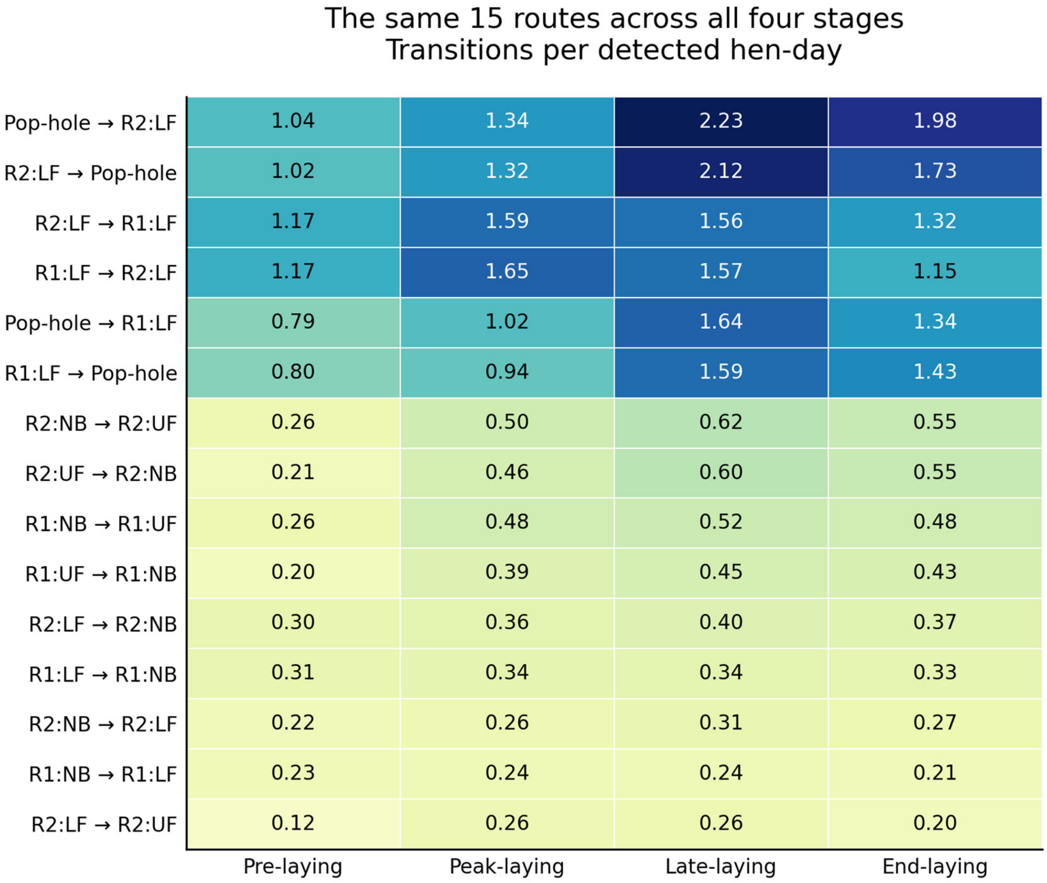

Figure S6. Numerical comparison of a fixed set of 15 non-looping routes. These same routes met the stage-specific 80th-percentile positive-count threshold in all four original full-network pooled comparisons. The fixed set is retained across columns; rates use original full-network detected hen-days. The figure does not imply equal detection probability or causal stage differences. Abbreviations follow Figure S1.

S8. Additional Robustness and Record Reconciliation

S8.1. Retained Dates and Age Reporting

The four names and nominal age bands follow Welch et al. (2023), cited as reference 19 in the main manuscript. Calendar windows and retained observations are reported separately. The production table matched 192 of 204 retained dates; 12 selected dates fell within its August–September gap. The project calendar documents

placement on 7 November 2016 at approximately 16 weeks and a nominal week-74 entry in December 2017. It contains no independently verified hatch date, and some other calendar week annotations differ.

The first available production row, 8 November 2016, recorded an age of 116 days. That date/age relation was shared by all 284 available rows through 18 August 2017. Extending it across calendar time was consistent with all 379 weekly-age labels when the label was treated as the ceiling of age in weeks. The age-in-days field advanced from 399 on 18 August to 400 on 2 September despite 15 elapsed days, leaving the subsequent daily counter 14 days behind calendar progression. The original file was preserved. This relation was used only to estimate the age coverage in Table S7, not to alter retained dates, stage membership, bouts or transitions.

The inferred reference date is a consistency parameter and is not asserted to be the hatch date. The weekly labels describe the week being entered under this inferred convention, rather than completed weeks; for example, 129 days corresponds to approximately 18.4 weeks and is labelled week 19. The estimates place all retained dates within their nominal completed-week bands, but do not imply observation throughout those bands. In particular, end-laying observations stop at approximately 69.7 weeks, rather than 74 weeks. Pre-laying light durations were shorter than the later 16-h schedule, so the fixed 04:00–19:59 audit window was retained as an operational recording rule, not relabelled as the actual light period on every date.

Table S7. Retained dates and approximate record-derived age coverage. Ages are estimates from the internally consistent date/age relation, not a verified hatch date. Matched production dates indicate the availability of a direct weekly-age entry.

Stage	Dates	First retained date	Last retained date	Estimated age (weeks)	Matched production dates
Pre-laying	14	2016-11-21	2016-12-15	18.4–21.9	14/14
Peak-laying	27	2017-01-05	2017-03-09	24.9–33.9	27/27
Late-laying	83	2017-03-16	2017-08-02	34.9–54.7	83/83
End-laying	80	2017-08-04	2017-11-15	55.0–69.7	68/80

S8.2. Cleaning Sensitivity Beyond Feeder Rankings

The joint route-sensitivity sample used five retained dates per stage and all allocation-valid hens before final-cohort restriction. The merge gap was held at 10 s while the minimum source-bout duration increased from 1 to 10 s. The strict non-feeder subset required both endpoints to be in R1:NB, R2:NB, Pop-hole or Range; all four indoor feeder nodes and RF were excluded. Nest-linked routes included at least one NB endpoint and could include a feeder at the other endpoint. Sum-of-absolute route changes prevents opposing route changes from cancelling.

Table S8. Counts and route changes in the 20-date cleaning sample at a fixed 10-s merge gap. Net changes are relative to counts at a 1-s minimum. The absolute-change column sums absolute changes for individual directed routes before dividing by the baseline subset total.

Stage	Route subset	Count at 1 s	Count at 10 s	Net change (%)	Absolute route change (%)
Pre-laying	All non-looping	129,791	129,289	-0.387	0.402
Pre-laying	Non-feeder	2,698	2,701	+0.111	0.111
Pre-laying	Nest-linked	34,635	34,275	-1.039	1.091
Peak-laying	All non-looping	170,315	169,810	-0.297	0.311
Peak-laying	Non-feeder	2,143	2,143	+0.000	0.187
Peak-laying	Nest-linked	46,763	46,423	-0.727	0.748
Late-laying	All non-looping	194,791	194,486	-0.157	0.177
Late-laying	Non-feeder	5,516	5,529	+0.236	0.236

Stage	Route subset	Count at 1 s	Count at 10 s	Net change (%)	Absolute route change (%)
Late-laying	Nest-linked	46,865	46,716	-0.318	0.373
End-laying	All non-looping	153,906	153,542	-0.237	0.272
End-laying	Non-feeder	5,544	5,555	+0.198	0.234
End-laying	Nest-linked	39,532	39,373	-0.402	0.458

Non-feeder total counts can increase slightly because removing a short intervening event can create a new adjacent pair within the selected endpoint subset. This is a sequence-construction consequence. Changing only the same-area merge gap leaves cross-area counts unchanged by construction, including in the non-feeder subset. These checks quantify sensitivity, not detection accuracy or behavioural truth.

S8.3. Cohort Coverage and Observed Heterogeneity

Individual hen-stage rates divide each hen's non-looping count by that hen's detected dates. Table S9A describes the 2,162 rates per stage; the quartiles are not confidence intervals. Table S9B compares primary rates with the same calculations in 1,480 hens meeting the ≥75% coverage rule in every stage. The stronger rule alters section composition and cannot correct sensor sensitivity.

Table S9. Individual-rate variability and higher-coverage cohort sensitivity. Rates are non-looping transitions per detected hen-day.

Panel A. Observed individual hen-stage rate distribution.

Stage	Hens	Q1	Median	Q3
Pre-laying	2162	6.14	9.93	12.93
Peak-laying	2162	11.07	13.56	16.04
Late-laying	2162	14.16	17.31	21.02
End-laying	2162	11.96	14.90	18.00

Panel B. Pooled rates in the primary and higher-coverage cohorts.

Stage	Primary cohort (2,162)	Coverage subset (1,480)	Relative change (%)
Pre-laying	9.65	9.37	-2.87
Peak-laying	13.54	13.34	-1.48
Late-laying	17.77	17.63	-0.77
End-laying	15.10	14.95	-0.94

S8.4. Within Day Gaps and Midnight Boundaries

The 204 cleaned daily files reconstructed 24,155,194 bouts, 398,488 detected hen-days and 23,756,706 transitions exactly. Daily counts also matched the original transition audit and the independent hen-day table. There were no identical sequence rows and no cleaned source bout whose exit extended beyond its entry date. This last finding describes the cleaned files; it does not establish whether events were split by the original recording software.

For the gap-cap analysis, original within-day adjacent pairs were filtered by nonnegative exit-to-next-entry gap and an upper limit of 15, 60 or 240 min, or no upper limit. No replacement adjacency was constructed. The no-upper-limit sensitivity excludes the 13 negative-gap cross-area pairs retained in the primary analysis. Rates use the same detected hen-days as the primary analysis; Table S10 gives counts, rates and percentage changes.

Table S10. Non-looping transition sensitivity to within-day gap limits. The no-upper-limit row excludes negative gaps; primary counts retain them. Limits are operational scenarios rather than validated movement thresholds.

Stage	Upper gap limit	Non-looping count	Rate	Change from primary (%)
Pre-laying	15 min	120,867	4.0251	-58.2735
Pre-laying	60 min	250,340	8.3369	-13.5760
Pre-laying	240 min	289,365	9.6365	-0.1036
Pre-laying	No upper limit	289,665	9.6465	0.0000
Peak-laying	15 min	368,563	6.4716	-52.2209
Peak-laying	60 min	703,931	12.3603	-8.7451
Peak-laying	240 min	771,056	13.5389	-0.0433
Peak-laying	No upper limit	771,390	13.5448	0.0000
Late-laying	15 min	1,604,490	9.6714	-45.5744
Late-laying	60 min	2,754,226	16.6017	-6.5744
Late-laying	240 min	2,946,788	17.7624	-0.0425
Late-laying	No upper limit	2,948,036	17.7700	-0.0002
End-laying	15 min	1,146,332	7.8727	-47.8506
End-laying	60 min	1,986,458	13.6424	-9.6312
End-laying	240 min	2,196,441	15.0845	-0.0786
End-laying	No upper limit	2,198,160	15.0963	-0.0004

A separate boundary analysis linked the final cleaned bout of one retained date to the first bout of the next date only when the dates were consecutive, in the same stage, and had the same hen and housing section. There were 170 eligible date interfaces and 33 nonconsecutive interfaces that were not bridged. Stage boundaries were not bridged. Added links had nonnegative gaps. Boundary limits applied only to those additions; existing primary within-day links remained unchanged.

Table S11. Cross-midnight candidate pairs and added non-looping links. Panel A counts candidate pairs before limiting gaps. Panel B gives added non-looping counts and percentage increases relative to each stage's primary count. Same-area candidate counts are not a rebuilt overnight loop analysis.

Panel A. Boundary candidates.

Stage	Consecutive date interfaces	Non-looping candidates	Same area gap ≤10 s	Same area gap >10 s
Pre-laying	11	2,163	0	21,450
Peak-laying	22	6,333	4	40,058
Late-laying	68	15,582	131	119,851
End-laying	69	19,548	87	105,502

Panel B. Added non-looping links, n (% of primary count).

Stage	≤15 min	≤60 min	≤240 min	No upper limit
Pre-laying	7 (0.0024%)	17 (0.0059%)	71 (0.0245%)	2,163 (0.7467%)
Peak-laying	10 (0.0013%)	30 (0.0039%)	171 (0.0222%)	6,333 (0.8210%)
Late-laying	20 (0.0007%)	50 (0.0017%)	156 (0.0053%)	15,582 (0.5286%)
End-laying	23 (0.0010%)	62 (0.0028%)	239 (0.0109%)	19,548 (0.8893%)

The boundary check counted 287,083 same-area candidates, including 222 gaps of at most 10 s. Rebuilding loops would require applying the merge rule across continuous dates, potentially changing bout boundaries. That reconstruction was not performed. The check consequently quantifies boundary sensitivity of recorded cross-area adjacency, not nocturnal occupancy, perching or the complete effect of segmentation on same-area records.

S8.5. Restrictions to Monitored Location Sets

Restrictions were applied to existing non-looping pairs. The six-indoor-node subset required both endpoints to be R1/R2 × LF/NB/UF. The second subset removed every pair with a Range or RF endpoint. Intervening records were not deleted and remaining bouts were not reconnected. Full-network detected hen-days stayed fixed, so these are restricted-link rates rather than exposure-matched or detection-corrected estimates.

Table S12. Non-looping rates under location restrictions, per original detected hen-day.

Stage	All non-looping links	Exclude Range/RF endpoints	Six indoor endpoints only
Pre-laying	9.6465	9.4481	5.5233
Peak-laying	13.5448	13.2862	8.2645
Late-laying	17.7700	16.7010	8.4917
End-laying	15.0964	14.0262	7.0201

The peak-to-late change was 31.2% in the full network and 2.7% for indoor-only links. The stage ordering was retained, but the magnitude changed. This cannot distinguish true changes around the pop-hole/outdoor interface from changes in recording coverage. Shared location labels do not guarantee equivalent antenna operation.

S8.6. Retained and Excluded Hens

Original metadata values were checked against the master workbook. The comparison includes 2,162 retained hens and 956 excluded hens with post-rearrangement assignments. Seven hens without assignments are not included in the latter group. Initial and 22-week weight labels follow the source records. Early pop-hole time is an inherited cumulative RFID detection summary, not an independently validated ranging phenotype or time continuously outdoors.

Table S13. Available characteristics in retained and excluded assigned hens. Entries give available n; median [Q1, Q3]; missing n. Missingness is reported separately and no missing values were imputed.

Characteristic	Retained hens (2,162)	Excluded assigned hens (956)
Weight at 16 weeks (kg)	n=2,160; 1.364 [1.298, 1.432]; missing 2	n=953; 1.364 [1.295, 1.428]; missing 3
Weight at 22 weeks (kg)	n=2,102; 1.760 [1.677, 1.845]; missing 60	n=643; 1.762 [1.665, 1.851]; missing 313
Early pop-hole detection time (min)	n=2,162; 89.08 [1.69, 300.83]; missing 0	n=956; 23.96 [0.18, 210.02]; missing 0

Initial weight medians were similar, but early recorded pop-hole histories and later weight availability differed. These comparisons show that stage-presence selection may affect representativeness; they do not establish mortality, tag loss or any other specific cause of exclusion.
