# Supplementary material for: Movement Patterns of Laying Hens Across Production Stages in a Commercial Aviary System
Source: Animals (Basel). 2026 Nov 9;16(21):3533. doi: 10.3390/ani16213533 (PMC13761489; doi:10.3390/ani16213533)
Supplement: Supplementary file 1 [file animals-16-03533-s001.zip › Supplementary_Interactive_Transitions/Supplementary_Interactive_Transitions.html]

Recorded hen transitions 

# Recorded transitions among monitored aviary areas

Supplementary interactive figures for Animals manuscript animals-4623964. These diagrams describe successive RFID detections in the 2,162-hen cohort. Select a stage, housing section and transition representation. Hover over a ribbon, or focus it with the keyboard, to inspect counts, rates and the detected hen-day denominator.

Housing sectionsPooled P1–P5P1P2P3P4P5Production stagePre-layingPeak-layingLate-layingEnd-layingRepresentationNon-looping transitionsLoop-inclusive transitions

Range†: regular streams P1/P4 only; RF is omitted from pooling and is available under P4.

Detected hen-days: **30,028**. The displayed routes meet the 80th percentile of positive route counts within this selection. Omitted routes need not have zero counts. Pooled diagrams omit RF; its routes use P4 denominators in the P4 selection.

OriginDestinationPop-hole → R1:LF | 23,846 recorded transitions | 0.794125 per detected hen-day | denominator 30,028Pop-hole → R2:LF | 31,289 recorded transitions | 1.041994 per detected hen-day | denominator 30,028R1:LF → Pop-hole | 24,145 recorded transitions | 0.804083 per detected hen-day | denominator 30,028R1:LF → R1:NB | 9,406 recorded transitions | 0.313241 per detected hen-day | denominator 30,028R1:LF → R2:LF | 34,992 recorded transitions | 1.165312 per detected hen-day | denominator 30,028R1:NB → R1:LF | 6,985 recorded transitions | 0.232616 per detected hen-day | denominator 30,028R1:NB → R1:UF | 7,856 recorded transitions | 0.261622 per detected hen-day | denominator 30,028R2:LF → Pop-hole | 30,505 recorded transitions | 1.015885 per detected hen-day | denominator 30,028R2:LF → R1:LF | 35,263 recorded transitions | 1.174337 per detected hen-day | denominator 30,028R2:LF → R2:NB | 9,149 recorded transitions | 0.304682 per detected hen-day | denominator 30,028R2:NB → R2:LF | 6,621 recorded transitions | 0.220494 per detected hen-day | denominator 30,028R2:NB → R2:UF | 7,881 recorded transitions | 0.262455 per detected hen-day | denominator 30,028R1:LF: displayed 2.282636 transitions/hen-day; all positive selected-representation routes 74,917 transitionsR1:LFR1:NB: displayed 0.494239 transitions/hen-day; all positive selected-representation routes 19,509 transitionsR1:NBR2:LF: displayed 2.494905 transitions/hen-day; all positive selected-representation routes 81,354 transitionsR2:LFR2:NB: displayed 0.482949 transitions/hen-day; all positive selected-representation routes 19,587 transitionsR2:NBPop-hole: displayed 1.836120 transitions/hen-day; all positive selected-representation routes 58,968 transitionsPop-holeR1:LF: displayed 2.201079 transitions/hen-day; all positive selected-representation routes 74,627 transitionsR1:LFR1:NB: displayed 0.313241 transitions/hen-day; all positive selected-representation routes 19,478 transitionsR1:NBR1:UF: displayed 0.261622 transitions/hen-day; all positive selected-representation routes 14,333 transitionsR1:UFR2:LF: displayed 2.427801 transitions/hen-day; all positive selected-representation routes 81,731 transitionsR2:LFR2:NB: displayed 0.304682 transitions/hen-day; all positive selected-representation routes 19,567 transitionsR2:NBR2:UF: displayed 0.262455 transitions/hen-day; all positive selected-representation routes 15,326 transitionsR2:UFPop-hole: displayed 1.819968 transitions/hen-day; all positive selected-representation routes 59,379 transitionsPop-holeRibbon widths are scaled separately for each selection. Use the rates for comparisons.

Left and right columns repeat location labels as origins and destinations. A same-area ribbon is a self-loop in the loop-inclusive representation. R1/R2 identify aviary rows; LF lower feeder; NB nest box; UF upper feeder; RF outdoor range feeder. Node sizes and ribbons represent recorded transitions, not occupancy or numbers of hens. A pop-hole link does not identify an inward or outward crossing. Hens were rearranged between pre- and peak-laying, so numbered sections changed composition.

## Displayed route counts and rates

Rates are transitions per detected hen-day. Widths are scaled independently across selections.

| Origin | Destination | Count | Rate |
| --- | --- | --- | --- |
| Pop-hole | R1:LF | 23,846 | 0.794125 |
| Pop-hole | R2:LF | 31,289 | 1.041994 |
| R1:LF | Pop-hole | 24,145 | 0.804083 |
| R1:LF | R1:NB | 9,406 | 0.313241 |
| R1:LF | R2:LF | 34,992 | 1.165312 |
| R1:NB | R1:LF | 6,985 | 0.232616 |
| R1:NB | R1:UF | 7,856 | 0.261622 |
| R2:LF | Pop-hole | 30,505 | 1.015885 |
| R2:LF | R1:LF | 35,263 | 1.174337 |
| R2:LF | R2:NB | 9,149 | 0.304682 |
| R2:NB | R2:LF | 6,621 | 0.220494 |
| R2:NB | R2:UF | 7,881 | 0.262455 |
